# Community health workers to improve uptake of maternal healthcare services: A cluster-randomized pragmatic trial in Dar es Salaam, Tanzania

**DOI:** 10.1371/journal.pmed.1002768

**Published:** 2019-03-29

**Authors:** Pascal Geldsetzer, Eric Mboggo, Elysia Larson, Irene Andrew Lema, Lucy Magesa, Lameck Machumi, Nzovu Ulenga, David Sando, Mary Mwanyika-Sando, Donna Spiegelman, Ester Mungure, Nan Li, Hellen Siril, Phares Mujinja, Helga Naburi, Guerino Chalamilla, Charles Kilewo, Anna Mia Ekström, Dawn Foster, Wafaie Fawzi, Till Bärnighausen

**Affiliations:** 1 Department of Global Health and Population, Harvard T.H. Chan School of Public Health, Boston, Massachusetts, United States of America; 2 Management and Development for Health, Dar es Salaam, Tanzania; 3 Department of Biostatistics, Harvard T.H. Chan School of Public Health, Boston, Massachusetts, United States of America; 4 Africa Academy for Public Health, Dar es Salaam, Tanzania; 5 Department of Epidemiology, Harvard T.H. Chan School of Public Health, Boston, Massachusetts, United States of America; 6 Department of Nutrition, Harvard T.H. Chan School of Public Health, Boston, Massachusetts, United States of America; 7 Department of Biostatistics, Center for Methods in Implementation and Prevention Science, Yale School of Public Health, Yale University, New Haven, Connecticut, United States of America; 8 Department of Behavioural Sciences, Muhimbili University of Health and Allied Sciences, Dar es Salaam, Tanzania; 9 Department of Public Health Sciences, Karolinska Institutet, Stockholm, Sweden; 10 Department of Pediatrics and Child Health, Muhimbili University of Health and Allied Sciences, Dar es Salaam, Tanzania; 11 Departments of Obstetrics and Gynaecology, Muhimbili University of Health and Allied Sciences, Dar es Salaam, Tanzania; 12 Department of Infectious Diseases, Karolinska University Hospital, Stockholm, Sweden; 13 Department of Psychiatry, Yale School of Medicine, Yale University, New Haven, Connecticut, United States of America; 14 Department of Social and Behavioral Sciences, Yale School of Public Health, Yale University, New Haven, Connecticut, United States of America; 15 Heidelberg Institute of Global Health, Heidelberg University, Heidelberg, Germany; 16 Africa Health Research Institute, Somkhele, KwaZulu-Natal, South Africa; University of California, San Francisco, UNITED STATES

## Abstract

**Background:**

Home delivery and late and infrequent attendance at antenatal care (ANC) are responsible for substantial avoidable maternal and pediatric morbidity and mortality in sub-Saharan Africa. This cluster-randomized trial aimed to determine the impact of a community health worker (CHW) intervention on the proportion of women who (i) visit ANC fewer than 4 times during their pregnancy and (ii) deliver at home.

**Methods and findings:**

As part of a 2-by-2 factorial design, we conducted a cluster-randomized trial of a home-based CHW intervention in 2 of 3 districts of Dar es Salaam from 18 June 2012 to 15 January 2014. Thirty-six wards (geographical areas) in the 2 districts were randomized to the CHW intervention, and 24 wards to the standard of care. In the standard-of-care arm, CHWs visited women enrolled in prevention of mother-to-child HIV transmission (PMTCT) care and provided information and counseling. The intervention arm included additional CHW supervision and the following additional CHW tasks, which were targeted at all pregnant women regardless of HIV status: (i) conducting home visits to identify pregnant women and refer them to ANC, (ii) counseling pregnant women on maternal health, and (iii) providing home visits to women who missed an ANC or PMTCT appointment. The primary endpoints of this trial were the proportion of pregnant women (i) not making at least 4 ANC visits and (ii) delivering at home. The outcomes were assessed through a population-based household survey at the end of the trial period. We did not collect data on adverse events. A random sample of 2,329 pregnant women and new mothers living in the study area were interviewed during home visits. At the time of the survey, the mean age of participants was 27.3 years, and 34.5% (804/2,329) were pregnant. The proportion of women who reported having attended fewer than 4 ANC visits did not differ significantly between the intervention and standard-of-care arms (59.1% versus 60.7%, respectively; risk ratio [RR]: 0.97; 95% CI: 0.82–1.15; *p =* 0.754). Similarly, the proportion reporting that they had attended ANC in the first trimester did not differ significantly between study arms. However, women in intervention wards were significantly less likely to report having delivered at home (3.9% versus 7.3%; RR: 0.54; 95% CI: 0.30–0.95; *p =* 0.034). Mixed-methods analyses of additional data collected as part of this trial suggest that an important reason for the lack of effect on ANC outcomes was the perceived high economic burden and inconvenience of attending ANC. The main limitations of this trial were that (i) the outcomes were ascertained through self-report, (ii) the study was stopped 4 months early due to a change in the standard of care in the other trial that was part of the 2-by-2 factorial design, and (iii) the sample size of the household survey was not prespecified.

**Conclusions:**

A home-based CHW intervention in urban Tanzania significantly reduced the proportion of women who reported having delivered at home, in an area that already has very high uptake of facility-based delivery. The intervention did not affect self-reported ANC attendance. Policy makers should consider piloting, evaluating, and scaling interventions to lessen the economic burden and inconvenience of ANC.

**Trial registration:**

ClinicalTrials.gov NCT01932138

## Abstract 2

In this cluster-randomized trial in Dar es Salaam, Tanzania, Pascal Geldsetzer & colleagues assessed how a community health worker intervention affected the proportion of women attending antenatal care and delivering their babies at home.

## Author summary

**Why was this study done?:**

- Along with raising the quality of maternal healthcare services, increasing uptake of antenatal care (ANC) and facility-based delivery is thought to be important to improve both maternal and neonatal health in resource-poor settings.
- While community health worker (CHW)–led interventions have been proposed to increase uptake of essential maternal care services, there is limited evidence for these interventions from large-scale randomized studies implemented in the routine health system.

**What did the researchers do and find?:**

- We randomized all 60 wards (geographic areas) in 2 of 3 districts of Dar es Salaam, Tanzania, to a CHW intervention—consisting of additional CHW supervision, CHW-led identification of newly pregnant women in the community, and CHW follow-up at home for those who missed an ANC or prevention of mother-to-child HIV transmission (PMTCT) appointment—or to the standard of care (counseling visits by CHWs to PMTCT patients only).
- We found no difference between study arms in self-reported ANC underutilization (attending ANC fewer than 4 times and not visiting ANC in the first trimester) but the intervention almost halved the probability of women reporting having delivered at home (3.9% in the intervention arm versus 7.3% in the standard-of-care arm).
- In a nested mixed-methods study, we found that a likely reason for the CHW intervention’s lack of effect on ANC utilization was that provision of information, counseling, and reminders by CHWs was insufficient to change women’s perception that the relatively high transport costs, lost time from work, and inconvenience of earlier and more frequent ANC attendance outweigh its benefits.

**What do these findings mean?:**

- The CHW intervention appears to have been effective in moving towards universal coverage of facility-based delivery in this population that already has high uptake of facility-based delivery. However, the CHW intervention was insufficient to improve the timeliness and frequency of ANC attendance.
- The main limitation of this study was that the outcomes were assessed through self-report only.
- Policy makers should consider piloting, evaluating, and scaling CHW interventions aimed at reducing the economic burden and inconvenience of ANC attendance, which could include shifting ANC tasks from nurses at healthcare facilities to CHWs in the community to decrease the number of required healthcare facility visits for ANC.

## Introduction

The World Health Organization (WHO) recommends frequent antenatal care (ANC) visits spaced at regular intervals during pregnancy [1]. A minimum of 4 ANC visits was recommended until 2016 [2], after which WHO changed its recommendation to a minimum of 8 visits [1], with the first visit taking place as early as possible and no later than the end of the first trimester. However, while almost all women in sub-Saharan Africa attend at least 1 ANC visit, few attend 4 or more visits, and few have their first visit within the first trimester [3]. This pattern is also true for Tanzania, where, according to the latest (2015–2016) Demographic and Health Survey, 98% of women had attended at least 1 ANC visit for their most recent live birth [4], but only 51% attended 4 ANC visits or more, and only 24% made their first visit during the first 3 months of pregnancy. Late and inconsistent ANC attendance may mean that health problems existing prior to pregnancy (e.g., sexually transmitted infections or anemia) or conditions arising during pregnancy (e.g., gestational diabetes or pre-eclampsia) are detected late, increasing the risk of adverse health outcomes for the mother and newborn [5]. In addition, the routine HIV testing conducted during ANC in sub-Saharan Africa generally serves as the entry point to prevention of mother-to-child HIV transmission (PMTCT) services [1,6]. In settings with high HIV prevalence, such as Tanzania [7], late and inconsistent ANC attendance is therefore likely to result in late initiation of antiretroviral therapy (ART) and poor ART adherence for pregnant women with HIV [6]. Improving the uptake of ANC is thus crucial to maximize the benefits of ART for both the mother and the newborn.

A second key maternal healthcare service recommended by WHO is birth attendance by skilled health personnel [8], which is interconnected with ANC uptake because promoting and planning for delivery in the presence of a skilled birth attendant is an important function of ANC [8]. The proportion of births that take place in the presence of a skilled birth attendant is an official indicator of the Sustainable Development Goals’ target for maternal mortality [9], and skilled attendance at birth is thought to be an essential intervention to reduce not only maternal deaths but also neonatal mortality and morbidity [10,11]. Tanzania’s Ministry of Health, Community Development, Gender, Elderly and Children aims to increase the proportion of births that take place at healthcare facilities—and thus are assumed to be taking place in the presence of a skilled birth attendant—from 50% in 2016 to 80% in 2020 [12].

WHO recommends the use of community health workers (CHWs) for increasing the uptake of ANC [13], PMTCT [14], and facility-based delivery [13]. Indeed, promoting the timely uptake of facility-based healthcare services, including ANC and PMTCT, is one of the core tasks of CHWs in low- and middle-income countries (LMICs) [15,16]. However, while the evidence from observational studies and performance evaluations of CHWs’ effectiveness in improving the uptake of maternal healthcare services is encouraging, WHO has noted that there is a strong need for large-scale randomized studies on this subject [16]. The few randomized trials identified by systematic reviews have suffered from potential biases and provided mixed results [16–20]. We report here the results of a large-scale cluster-randomized pragmatic implementation trial of a CHW program for increasing uptake of maternal healthcare services. More specifically, this trial aimed to determine the causal impact of additional supervision and maternal healthcare tasks for Dar es Salaam’s public-sector CHW program on the proportion of pregnant women who (i) attend fewer than 4 ANC visits and (ii) deliver at home.

## Methods

### Study setting

The study was carried out in 2 (Kinondoni District and Ilala District) of 3 districts of the Dar es Salaam region in Tanzania, which is Tanzania’s most urban region. The study districts had 4.4 million inhabitants in 2012, which corresponded to 69% of Dar es Salaam’s total population in that year [21].

### Study design

The detailed protocol for this trial has been published elsewhere [22]. The CONSORT checklist for pragmatic trials for this report can be found in S1 Checklist. This study was a cluster-randomized pragmatic trial with a 2-by-2 factorial design, with the second randomization being for a trial of 2 different medication regimens—WHO Option A and Option B—for PMTCT. Specifically, WHO Option A consisted of zidovudine from 14 weeks of gestation until delivery for the mother, and daily nevirapine until 1 week after stopping breastfeeding for the infant (or either daily nevirapine or a single dose of nevirapine plus daily zidovudine for 4 to 6 weeks after delivery if the infant was not breastfed). Option B consisted of 1 of 3 triple antiretroviral drug regimens from 14 weeks’ gestation until 1 week after stopping breastfeeding for the mother, and nevirapine plus zidovudine for 4 to 6 weeks after delivery for the infant. The primary outcome measures of the trial of WHO Option A versus B were the proportion of (i) infants born to mothers living with HIV who had acquired HIV, (ii) HIV-exposed infants tested for HIV, and (iii) HIV-positive women receiving PMTCT, which will be reported elsewhere. This paper focuses on the trial of the CHW intervention versus the standard of care. The unit of randomization was a ward, which is the administrative unit below a district in Dar es Salaam.

### Study duration

This trial was conducted over a period of 19 months from 18 June 2012 to 15 January 2014. The trial was stopped 4 months early due to the decision by Tanzania’s Ministry of Health, Community Development, Gender, Elderly and Children to adopt Option B+ for PMTCT. The introduction of Option B+ changed the standard of care for PMTCT in Tanzania (which had been Option A at the time of trial start) to a PMTCT option that WHO judged to be superior to Option A and B [6]. The continuation of the trial after this change would thus have been unethical.

### Randomization and masking

The 2 study districts consisted of 60 wards, which are the administrative unit above the neighborhood level in Dar es Salaam. Wards were block-randomized within the following strata that were expected to be predictive of the outcome: district (Ilala and Kinondoni) and type of healthcare facility (military, facility able to carry out cesarean sections, or facility unable to carry out cesarean sections). Within each stratum, wards were sorted by the expected number of pregnancies in the ward. After each round of randomizing 1 ward to each stratum, the assignment probabilities were revised to optimize balance in the expected number of pregnancies over the study period across study arms. The randomization was conducted by the study statistician (DS) and Ellen Hertzmark. Thirty-six wards were randomized to the CHW intervention and 24 to the standard of care. Study participants, nurses, and CHWs were not blinded to the assigned intervention because it would have been impossible to blind participants to whether they received a CHW visit, and healthcare workers to whether they were asked to carry out activities that they did not have to conduct previously.

### The standard of care

In the standard-of-care wards, this trial did not interfere with the usual functioning of the health system. Specifically, each neighborhood (“mtaa” in Swahili) had between 1 and 3 CHWs; CHWs were assigned to work in the neighborhood in which they also lived. The CHWs (referred to as home-based carers, or HBCs, in Tanzania) are a lay health worker cadre employed by the government. To be eligible to become a CHW, a person must have at least completed primary school. In the study areas, most CHWs (81%) were women, and three-quarters (74%) were aged between 30 and 49 years old. Their renumeration was a monthly stipend of US$35 (equivalent to approximately 63 purchasing power parity-adjusted international dollars [int$]). Existing since 1996, the CHW program had approximately 35,000 CHWs across Tanzania in 2015. All CHWs in Dar es Salaam were affiliated with 1 particular healthcare facility, which was located in or near to their assigned neighborhood. At each of these healthcare facilities, 1 nurse (henceforth referred to as the “community outreach nurse”) was responsible for supervising all CHWs (between 2 and 5 CHWs, depending on the facility) affiliated with that healthcare facility. At the beginning of the study period, there were 20 community outreach nurses and 157 CHWs working in the standard-of-care wards.

CHWs in the standard-of-care wards were responsible for providing services to patients living with HIV—encompassing pre-ART, ART, and PMTCT patients—who attended the healthcare facility with which the CHW was affiliated and who resided in the CHW’s neighborhood. Specifically, the CHWs were responsible for visiting these patients at home at least once every 3 months to provide counseling on ART adherence, family planning, and nutrition; promote the uptake of preventive healthcare services, such as vaccination for children; and refer unwell household members to the nearest healthcare facility. We used data from the ANC registration books of all healthcare facilities in the study areas for 1 month in 2011 to estimate the proportion of pregnancies in which the pregnant woman was HIV-positive (either from the beginning of or starting during the pregnancy). Given that almost all (96% in 2010 and 98% in 2015–2016 [4,23]) pregnant women in Tanzania visit ANC at least once, this method is unlikely to have missed a substantial number of pregnancies in the study areas. Based on these data, we estimated that 7% of all pregnancies during the study period were among women living with HIV. Thus, unlike in the intervention wards, the CHW activities in the standard-of-care wards targeted only a minority of all pregnant women.

At healthcare facilities in the standard-of-care wards, PMTCT patients who were lost to follow-up—defined as being more than 7 days late for an appointment—were called by telephone by the community outreach nurse. If the nurse was unable to contact the patient by phone, she/he was responsible for visiting the patient at home. However, during the trial preparation phase, the community outreach nurses informed the study team that these home visits rarely take place because they are time-consuming and nurses are not reimbursed for any travel expenses. We did not provide any training for CHWs or community outreach nurses for this trial in the standard-of-care wards.

### The CHW intervention

As part of the CHW intervention, 31 community outreach nurses were added to an already existing 21 community outreach nurses in the intervention wards (which we refer to as “additional supervision” in this paper), and 52 CHWs were added to an already existing 163 CHWs in neighborhoods in intervention wards that had a higher than average number of expected pregnancies during the study period. Both CHWs and community outreach nurses were tasked with additional maternal healthcare tasks (detailed below). No additional payment was provided to CHWs in the intervention wards. Each community outreach nurse in the intervention wards received a salary supplement of US$49 (approximately int$88) per month as compensation for additional work responsibilities arising from the CHW intervention.

CHWs in the intervention wards were responsible for carrying out 4 additional maternal healthcare tasks. First, they were responsible for identifying newly pregnant women through household visits and referring them to ANC using a referral card. Second, CHWs revisited pregnant women at home to verify whether they had attended ANC. Third, CHWs provided education and counseling to all pregnant women (as opposed to only PMTCT patients in the standard-of-care wards) and their household members on the importance of ANC, the benefits of partner involvement in ANC, facility-based delivery, HIV testing during pregnancy, PMTCT, breastfeeding, and postnatal care, and on more general health topics (e.g., nutrition and sanitation). Fourth, CHWs visited women at home who missed an ANC or PMTCT appointment to encourage them to attend.

The fourth point in the list above—visiting those at home who had missed their ANC or PMTCT appointment—was implemented in collaboration with the community outreach nurses. Specifically, “map cues” were taken by nurses during the first ANC visit, which consisted of the woman’s address and a written description of where and how her home could be found as well as the woman’s cell phone number. Because not all healthcare facilities that provided ANC in Dar es Salaam had a community outreach nurse, the community outreach nurses visited all ANC facilities in their ward on a weekly basis to collect the map cues of all women who were more than 7 days late for their ANC or PMTCT appointment. The community outreach nurses then tried to reach these patients by telephone to make a new appointment for them. If they could not reach the patient by phone, the map cue was passed on to the CHW who worked in the neighborhood in which the patient resided. In addition to collecting and passing along the map cues of those who were late for their appointment, the community outreach nurses compared the list kept by CHWs of whom they referred to ANC with the referral forms obtained from women at the ANC facilities. If women appeared on the CHWs’ lists but there was no matching referral card at the ANC facility, the community outreach nurse concluded that these women may not have attended ANC despite the referral. The community outreach nurse informed the CHWs of these women, who were then visited again at home by the CHWs to encourage them to attend ANC. These 2 tasks—collecting map cues from ANC facilities and comparing CHWs’ referral lists to the referral cards at healthcare facilities—were the only 2 additional tasks carried out by community outreach nurses in the intervention wards that were not carried out in the standard-of-care wards.

We provided 5 types of training for CHWs and community outreach nurses in the intervention wards. At the beginning of the study period, we conducted a 5-day training for 215 CHWs and 54 community outreach nurses in the intervention wards in all intervention activities, a 2-day training for 54 community outreach nurses in the use of a monitoring and evaluation tool for the CHW intervention, and a 3-day training for 213 CHWs in the use of the monitoring and evaluation tool. During the study period, we additionally provided a 3-day refresher training for 141 CHWs (all 215 CHWs in the intervention wards were invited but only 141 attended) and a 2-day refresher training for 54 community outreach nurses. These trainings were carried out by Management and Development for Health (MDH), which is a Tanzanian organization located in Dar es Salaam and working in partnership with Tanzania’s Ministry of Health, Community Development, Gender, Elderly and Children. In addition, the CHW program manager held a monthly meeting with all CHWs in the intervention wards during which CHWs could discuss any challenges that they faced in the field.

Because this was a pragmatic trial, we designed the intervention as closely as possible to the way that it would be implemented if it were rolled out by the government without an accompanying impact evaluation. As such, the performance of CHWs was monitored by the community outreach nurses, who are the CHWs’ supervisors in the routine health system. Thus, if a CHW performed below expectations, it was the responsibility of the community outreach nurse to identify the problem and find a solution. As per the standard protocols in the Dar es Salaam health system, community outreach nurses could inform the CHW program manager of any problems they encountered with a CHW. The performance of the community outreach nurses in turn was monitored by the chief nurse at each healthcare facility. Nonetheless, the study team did monitor CHWs’ activities in the intervention wards by requiring them to keep a register in which they logged each household visit to a pregnant woman. We present data from these registers in the Results section. The study team decided against more intense monitoring of the intervention activities to avoid interfering with the usual functioning of the health system.

### Theory of change

We have described the theory of change for this intervention in detail elsewhere [24]. In brief, we expected that the CHW home visits would increase uptake of ANC and facility-based delivery through (i) counseling, which raises awareness of ANC and can provide a source of motivation; (ii) informing women of the location of the nearest healthcare facility that provides ANC; (iii) the visit itself serving as a reminder or “nudge” to women who were already planning to attend ANC or deliver at a healthcare facility; and (iv) CHWs exerting a normative social influence on women through their visits and by emphasizing the importance of ANC and facility-based delivery. We hypothesized that these activities would lead to an increase in the proportion of women who delivered at a healthcare facility and an improvement in the timeliness and frequency of ANC uptake as compared with the standard-of-care wards because the activities of CHWs and community outreach nurses in the standard-of-care wards focused on only a subset of pregnant women (those living with HIV), CHW activities did not include efforts to identify and counsel newly pregnant women prior to their first ANC/PMTCT visit, and community outreach nurses did not instruct CHWs to follow up with women who missed an ANC/PMTCT appointment.

### Eligibility criteria

All pregnant women who were identified in the community by a CHW or who attended ANC or PMTCT at one of the healthcare facilities in the 2 study districts were eligible to be enrolled into the study. Women were enrolled continuously during the study period (June 2012 to January 2014) by clinicians during ANC visits and by CHWs during home visits.

### Endpoints

As stated in the study protocol [22], the trial of the CHW intervention had 2 primary endpoints: (i) the proportion of pregnant women attending fewer than 4 ANC visits and (ii) the proportion of pregnant women delivering at home. The trial also had 1 secondary endpoint: the proportion of pregnant women who did not attend their first ANC visit during the first trimester. These outcomes were selected in consultation with Tanzania’s Ministry of Health, Community Development, Gender, Elderly and Children because they were of direct interest to local and national policy makers to guide decisions on future developments of CHW programs. The 3 additional primary endpoints registered on ClinicalTrials.gov for this study—the proportion of infants born to HIV-infected mothers who had acquired HIV, the proportion of HIV-exposed infants tested for HIV, and the proportion of HIV-positive women receiving PMTCT—pertained only to the randomization to WHO Option A versus WHO Option B for PMTCT as part of the 2-by-2 factorial design of this trial. We also show results for the proportion never attending ANC during the pregnancy, which is an outcome that was not prespecified in our study protocol.

### Endpoint assessment

As a pragmatic trial, we aimed to minimize data collection activities that could interfere with the normal functioning of the health system. As described in our study protocol [22], we therefore intended to collect data for all endpoints in this trial from routine clinical registers in the public-sector health system. However, due to data quality concerns and difficulties in linking data for individual patients across the multiple clinical registers in use in the public healthcare system, we additionally conducted a population-based household survey at the end of the trial period to ascertain the trial’s endpoints. This survey was conducted from May 2014 to August 2014. The survey employed 2-stage cluster random sampling, whereby 122 (out of 168) neighborhoods in the intervention wards and 58 (out of 107) neighborhoods in the standard-of-care wards were randomly selected in the first stage. After beginning trial implementation, 6 wards in the standard-of-care arm were found to not have a functional healthcare facility that provided ANC or delivery services. These wards were excluded from the household survey because they may not have been comparable to those with a functioning healthcare facility. In addition to the fact that only 24 wards were randomized to the standard-of-care arm (as opposed to 36 to the intervention arm), this exclusion was responsible for the lower number of neighborhoods selected for the household survey in the standard-of-care wards than in the intervention wards. The second stage was a systematic random sample of 60 households in each neighborhood. Specifically, within each neighborhood, the survey team used a random number generator to select the first household, after which every fifth household in a randomly selected direction from the first household was sampled until a total of 60 households was contacted. We anticipated that sampling the same number of households in each neighborhood would be most efficient for the CHWs, who acted as data collectors for the household survey. To minimize biases, CHWs collected data in neighborhoods in which they did not normally work. In each of the households visited, the CHW asked whether any of the household members had been pregnant at any time during the study period. If so, the CHW administered a questionnaire to the woman who had been pregnant, which asked about maternal health knowledge, ANC attendance, and place of delivery. The questionnaire is provided in S1 Text. Due to feasibility constraints, households were not revisited if no one was present at the time of the CHW visit.

### Power

Based on projections of population size from the 2012 Tanzania census and the observed number of pregnancies in clinical registers [21], we assumed that a data collector would visit a mean of 5 households to identify 1 woman who was pregnant during the trial period. Given the sampling strategy for the household survey described above, we therefore expected a sample size of 1,464 women in intervention wards and 696 women in standard-of-care wards. Based on the results from the most recent Tanzania HIV/AIDS and Malaria Indicator Survey (at the beginning of the study period) [25], we assumed that 57% of participants in the standard-of-care wards would make fewer than 4 ANC visits, and that 10% would deliver at home. With a type I error rate of 0.05, the household survey had 80% power to detect a minimum difference in each of the 2 primary endpoints of 5 percentage points between standard-of-care and intervention wards. This minimally important difference was determined in consultation with the local municipal government and the Tanzanian Ministry of Health and Social Welfare. The calculation assumed unequal cluster sizes using the formula given by Donner and Klar [26], and an intracluster correlation coefficient (ICC) of 0.09, which was based on ICCs calculated using data from clinical registers at the healthcare facilities in the study area. In our registered study protocol [22], we show a sample size calculation based on the assumption that we would be able to use the health system’s routine clinical registers to assess our endpoints. The choice to instead conduct a household survey at the end of the study period was made because it became evident during the study period that women could not be reliably matched across the multiple clinical registers used at healthcare facilities. Therefore, the sample size calculation for this household survey was not prespecified in our study protocol. This change only affected the sample size for the outcome assessment; the number of administrative units (wards) that were randomized remained the same as registered in our protocol. This trial had no stopping rules because the intervention was deemed to have a minimal potential for adverse effects.

### Assessment of possible reasons for the observed effects

We found that the intervention did not have a significant effect on the ANC endpoints. To generate insights into the reasons for this observed lack of effect, we analyzed 3 additional data sources: (i) a patient register in which the CHWs logged each household visit they had made to a pregnant woman in their neighborhood as part of the study, (ii) an interviewer-administered questionnaire among PMTCT patients at healthcare facilities in the study area, and (iii) semi-structured qualitative interviews with community outreach nurses, CHWs, and patients. None of these analyses were prespecified in our study protocol and thus should be interpreted as being only exploratory in nature. For these exploratory analyses, we show all analyses that we conducted either in the Results section or in S2–S5 Figs and S6 Table. Relevant data from the 3 sources are presented under 4 broad possible reasons that the study team hypothesized may have led to the lack of effect on ANC outcomes: (i) the quantity of CHW visits was insufficient, (ii) the quality of CHW visits was insufficient, (iii) poor ANC quality discouraged ANC attendance, and (iv) the economic burden and/or inconvenience of attending ANC discouraged attendance.

#### Survey among PMTCT patients

The survey among PMTCT patients was conducted in March and April 2014 at 36 healthcare facilities in the study area. While PMTCT patients were not the focus of the CHW intervention, we present data from this questionnaire because PMTCT care at these healthcare facilities was provided by the same clinicians who provided ANC and is thus likely to be reflective of the quality of ANC. Similarly, the economic obstacles to attendance of PMTCT care faced by patients in the study area are likely to be similar to those for attending ANC. In March 2014, 150 healthcare facilities in the 2 study districts provided PMTCT care (49 in Ilala and 101 in Kinondoni). Of these, 92 healthcare facilities were excluded from our study because they were not public-sector facilities and thus did not have a community outreach nurse and affiliated CHWs. A further 10 facilities were excluded because the study team was unable to gain permission from the healthcare facility’s leadership to sample patients for the survey. After these exclusions, 18 healthcare facilities remained in the Ilala district, of which all were sampled. We then selected a further 18 facilities from the Kinondoni district to match the sampled facilities in Ilala—within 3 strata of healthcare facility type (dispensary, health center, or hospital)—as closely as possible in terms of PMTCT patient volume; 30 dispensaries, 3 health centers, and 3 hospitals were included in the survey. On a random set of data collection days, we selected a simple random sample of 3 PMTCT patients for the questionnaire from the list of all patients who had an appointment for PMTCT care on that day at the given healthcare facility. These patients were approached by an interviewer after their PMTCT visit for an interviewer-administered questionnaire. The questionnaire (shown in S2 Text) was administered in Swahili. Participants received TSh 5,000 (int$8.31) to compensate them for their time; participants were only informed of this compensation after providing consent for participation. A total of 595 patients participated. Because smaller healthcare facilities often had fewer than 3 PMTCT patients on a given data collection day, the number of patients sampled per facility varied from 1 to 64, with a mean of 16.1 (standard deviation: 15.0) and a median of 11.0 (interquartile range [IQR]: 4.0–22.0) participants. The sample characteristics of these patients are presented in S1 Table. In the Results section, we present unweighted descriptive statistics from this questionnaire whereby standard errors were adjusted for clustering at the level of the healthcare facility.

#### Qualitative interviews

The semi-structured qualitative interviews for our nested mixed-methods study were conducted with a purposive sample of 5 community outreach nurses from the CHW intervention wards, 5 CHWs from the intervention wards, and 10 pregnant women. The aim of these interviews was to characterize the views of community outreach nurses, CHWs, and patients regarding the CHW intervention and the quality of ANC and PMTCT care. The interview participants were selected to provide representation across age groups and, for the community outreach nurses and CHWs, number of years of experience in their job. Participants were selected at 5 healthcare facilities (3 dispensaries, 1 health center, and 1 hospital), all of which offered both ANC and PMTCT services. The participating pregnant women were interviewed after attending either ANC or PMTCT care at these facilities. The interviews took place in a private room and were taped using an audio recorder. All interviews were conducted in Swahili, by the same interviewer, and transcribed immediately after the interview into Microsoft Word. HS analyzed the transcripts using conventional content analysis [27]. Thus, codes for views on the CHW intervention and ANC and PMTCT care were developed directly from the data, rather than being defined prior to data analysis based on theory. The data collection for the nested mixed-methods study—which uses this qualitative analysis jointly with descriptive quantitative data—was conducted synchronously to the trial. The mixed-methods analyses served an explanatory purpose: to elucidate the contextual conditions and potential mechanisms leading to our trial results [28,29].

### Statistical analysis of the trial endpoints

Employing an intent-to-treat approach, we compared the proportion of women with the outcome of interest between the intervention and standard-of-care wards regardless of whether women reported having been visited by a CHW. Because of difficulty in ascertaining household survey participants’ migration patterns, for the analyses on ANC uptake we included all women who were resident in the study area at the time of the household survey and who reported having been pregnant for any amount of time during the study period, and for the analyses on uptake of facility-based delivery we excluded only those women who had not yet delivered at the time of the survey. Log-binomial regressions were used to determine the risk ratio (RR), which compares the proportion of women who had the outcome of interest between the 2 study groups. We chose to express our outcomes in the negative direction (e.g., did *not* deliver at a healthcare facility) to emphasize the intervention’s focus on achieving coverage in the relatively small proportion of pregnant women in urban Tanzania who are not yet covered by essential maternal healthcare services. An additional advantage is that by coding the outcomes as uncommon events, the RR is similar in magnitude to the odds ratio, which we obtained from logistic regressions as robustness checks. We employed the same logic for maternal health knowledge, which we also expected to be comparatively high among this population. We used heteroscedasticity-consistent (robust) standard errors, which we adjusted for clustering at the level of the unit of randomization (i.e., the ward). This was a complete case analysis. Despite having 2 primary endpoints, we did not adjust for multiple hypothesis testing because Tanzania’s Ministry of Health, Community Development, Gender, Elderly and Children clarified prior to the trial that the intervention would need to have a beneficial effect on both primary endpoints to be considered a success, and thus to be scaled up to other areas. ICC values for each outcome were calculated using the variance components from a 1-way analysis of variance (ANOVA) with 95% confidence intervals (CIs) computed with the exact method proposed by Thomas and Hultquist [30] and Donner [31] for unbalanced data. The statistical analysis was conducted in Stata 13.1.

Our prespecified analysis plan assumed that we would be able to use data from clinical registers—as opposed to the population-based survey—as our primary data source [22]. We nonetheless adhered to the prespecified analysis plan as closely as possible by using robust clustered log-binomial models, with the primary analyses not adjusting for any covariates (including the randomization to PMTCT Option A versus Option B). However, in secondary analyses, we show the results when adjusting for 5-year age group and a binary indicator for having completed secondary school or a tertiary education, and when using logistic regression models instead of log-binomial models.

### Ethical approval

This trial was approved by the National Institute of Medical Research in Tanzania (protocol number: NIMR/HQ/R.8a/Vol.IX/1351) in June 2012, and by the institutional review board of the Harvard T.H. Chan School of Public Health (protocol number: 22802) in January 2013.

## Results

**Fig 1:**
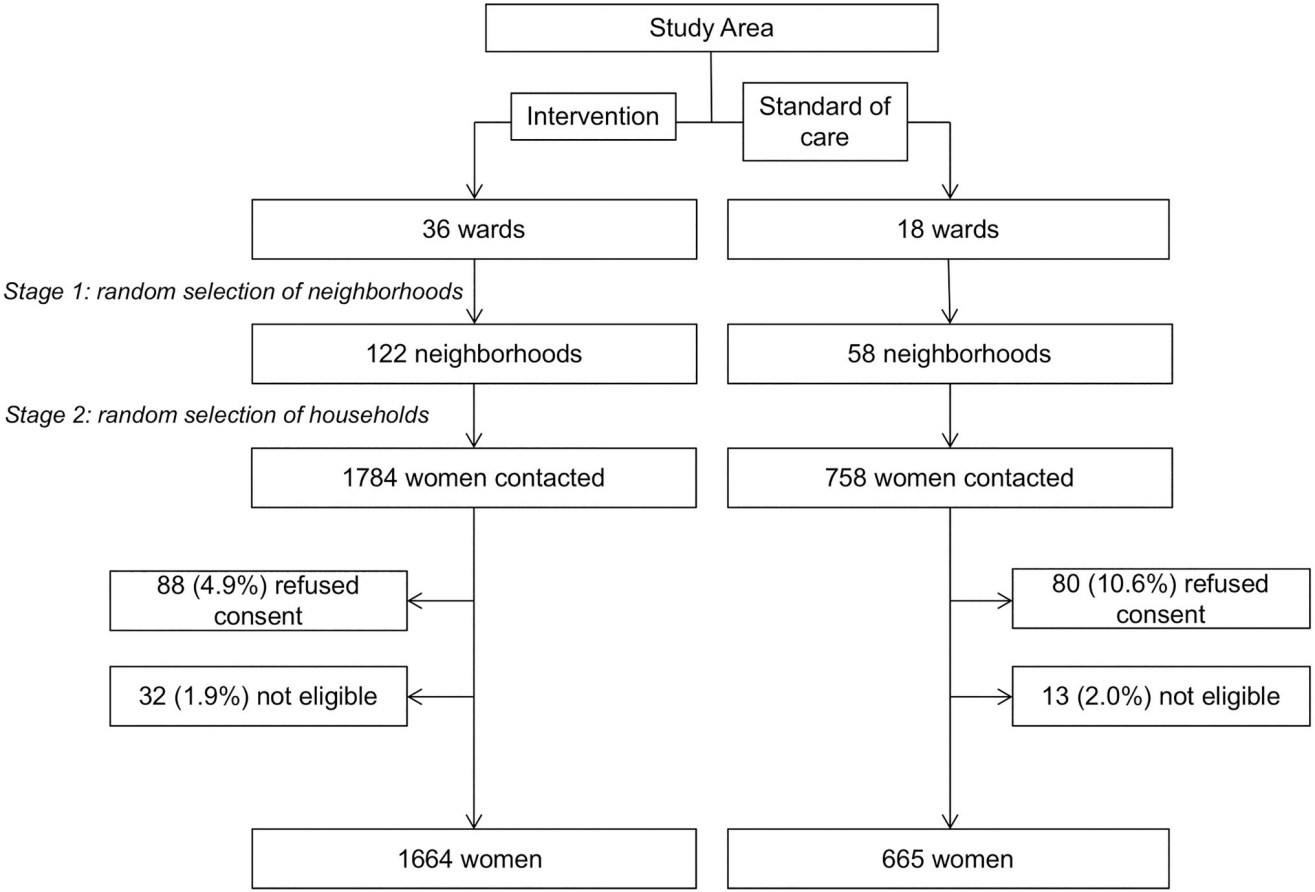
Selection of participants for the household survey. Women were deemed ineligible for study entry if they neither were currently pregnant nor had delivered a child within the previous 2 years (from June 2012 to May 2014).

A total of 7,320 and 3,480 households were visited, and 1,784 and 758 women contacted, in the household survey in the intervention and standard-of-care wards, respectively (Fig 1). In all, 1,664 women in the intervention wards and 665 women in the standard-of-care wards were interviewed, which is similar to our projected sample size (1,464 and 696 women, respectively). The mean age of the participants was 27.3 (SD: 5.9) years, and 34.5% (804/2,329) of the women were pregnant at the time of the household visit. For 59 (8.9%) and 114 (6.9%) participants in the standard-of-care and intervention wards, respectively, we could not ascertain from the questionnaire data whether the participant was currently pregnant or had recently delivered. These participants were included in all analyses; we provide results in S4 Table for analyses excluding these participants. The participant characteristics were well balanced across the 2 study arms (Table 1).

**Table 1:** Participant characteristics^1^.

Characteristic	Intervention (*n =* 1,664)	Standard of care (*n =* 665)
Age, mean (SD), years	27.3 (5.9)	27.4 (5.9)
Age group, *n* (%)		
15–19 years	107 (6.7%)	49 (7.7%)
20–24 years	464 (29.1%)	176 (27.6%)
25–29 years	467 (29.3%)	179 (28.1%)
30–34 years	361 (22.6%)	153 (24.0%)
35–39 years	145 (9.1%)	63 (9.9%)
≥40 years	52 (3.3%)	17 (2.7%)
Educational attainment, *n* (%)		
Less than primary	103 (6.6%)	55 (8.9%)
Primary	941 (60.2%)	363 (58.7%)
Secondary	482 (30.9%)	176 (28.5%)
More than secondary	36 (2.3%)	24 (3.9%)
Number of household members, mean (SD)		
≥18 years	2.8 (1.1)	2.7 (1.1)
<18 years	2.0 (1.4)	2.1 (1.5)
Pregnant, *n* (%)	559 (33.6%)	245 (36.8%)
Recently delivered^2^, *n* (%)	991 (59.6%)	361 (54.3%)
Number of days since delivery^3^, mean (SD)	274.0 (201.2)	270.1 (190.5)

^1^None of the means and proportions differ significantly between the 2 study arms.

^2^This is the number of women who delivered within the previous 2 years and were not currently pregnant.

^3^This number excludes women who were currently pregnant.

### Effect of the intervention on prespecified trial endpoints

Among those who should have answered each of the questions used to assess the outcomes (2,329 women for ANC outcomes, 1,525 women for place of delivery, and 865 women for intended place of delivery), 9.9% (151/1,525; 8.8% [37/420] in standard-of-care wards and 10.3% [114/1,105] in intervention wards) of observations were missing for place of delivery, 7.5% (65/865; 6.1% [16/263] in standard-of-care wards and 8.1% [49/602] in intervention wards) for intended place of delivery, 8.3% (194/2,329; 10.5% [70/665] in standard-of-care wards and 7.5% [124/1,664] in intervention wards) for not attending ANC in the first trimester, 15.0% (350/2,329; 12.3% [82/665] in standard-of-care wards and 16.1% [268/1,664] in intervention wards) for attending ANC fewer than 4 times, and 2.2% (50/2,329; 2.1% [14/665] in standard-of-care wards and 2.2% [36/1,664] in intervention wards) for never attending ANC.

There was no difference between study arms in the proportion of women visiting ANC fewer than 4 times and the proportion not attending the first ANC visit in the first trimester (Table 2). Slightly fewer women reported having never attended ANC during their pregnancy in the intervention than the standard-of-care arm, but the difference was small and insignificant (5.0% versus 6.1% in the standard-of-care and intervention arm, respectively; RR: 0.81; 95% CI: 0.36–1.80; *p =* 0.605). The results did not change significantly when disaggregating the study population by whether women had completed 9 months of pregnancy at the time of the household survey (S2 Table).

**Table 2:** Place of delivery and ANC attendance by study arm^1^.

Outcome	Intervention (%)	Standard of care (%)	Risk ratio (95% CI)	*p*-Value
**Delivery**				
Delivered at home^2^ (*n =* 1,374)	3.9	7.3	0.54 (0.30–0.95)	0.034
Does **not** intend to deliver in a healthcare facility^3^ (*n =* 800)	1.3	3.6	0.35 (0.13–0.94)	0.038
**ANC attendance**^4^				
Attended ANC <4 times (*n =* 1,979)	59.1	60.7	0.97 (0.82–1.15)	0.754
Did not attend ANC in first trimester (*n =* 2,135)	69.7	70.3	0.99 (0.87–1.13)	0.910
Never attended ANC (*n =* 2,279)	5.0	6.1	0.81 (0.36–1.80)	0.605

^1^Standard errors were adjusted for clustering at the ward level.

^2^This question was asked only to women who had delivered within the previous 2 years.

^3^This question was asked only to currently pregnant women.

^4^During the current pregnancy (for currently pregnant women) or the most recent pregnancy (for women who delivered within the previous 2 years).

ANC, antenatal care.

Fewer women who gave birth during the study period reported having delivered at home in the intervention wards compared to standard-of-care wards (RR: 0.54; 95% CI: 0.30–0.95). However, the absolute difference (3.9% versus 7.3% in intervention and standard-of-care wards, respectively) was small. Among women who were pregnant at the time of the household survey, the intention not to deliver in a healthcare facility was also lower in the intervention than standard-of-care wards (RR: 0.35; 95% CI: 0.13–0.94).

In S3–S5 Tables, we show all results when adjusting for age group and educational attainment (S3 Table), when excluding those women for whom the questionnaire data did not allow us to determine whether they were currently pregnant or had recently delivered (S4 Table), and when using logistic regression instead of log-binomial models (S5 Table). The point estimates obtained in these supplementary analyses are similar to the ones shown in Table 2.

The ICC values—with a cluster being a ward—were 0.036 (95% CI: 0.014–0.072) for having delivered at home, 0.109 (95% CI: 0.072–0.168) for attending ANC fewer than 4 times, and 0.060 (95% CI: 0.036–0.100) for not attending ANC within the first trimester.

### Possible reasons for the intervention’s failure to affect ANC outcomes

#### Was the quantity of CHW visits insufficient?

**Fig 2:**
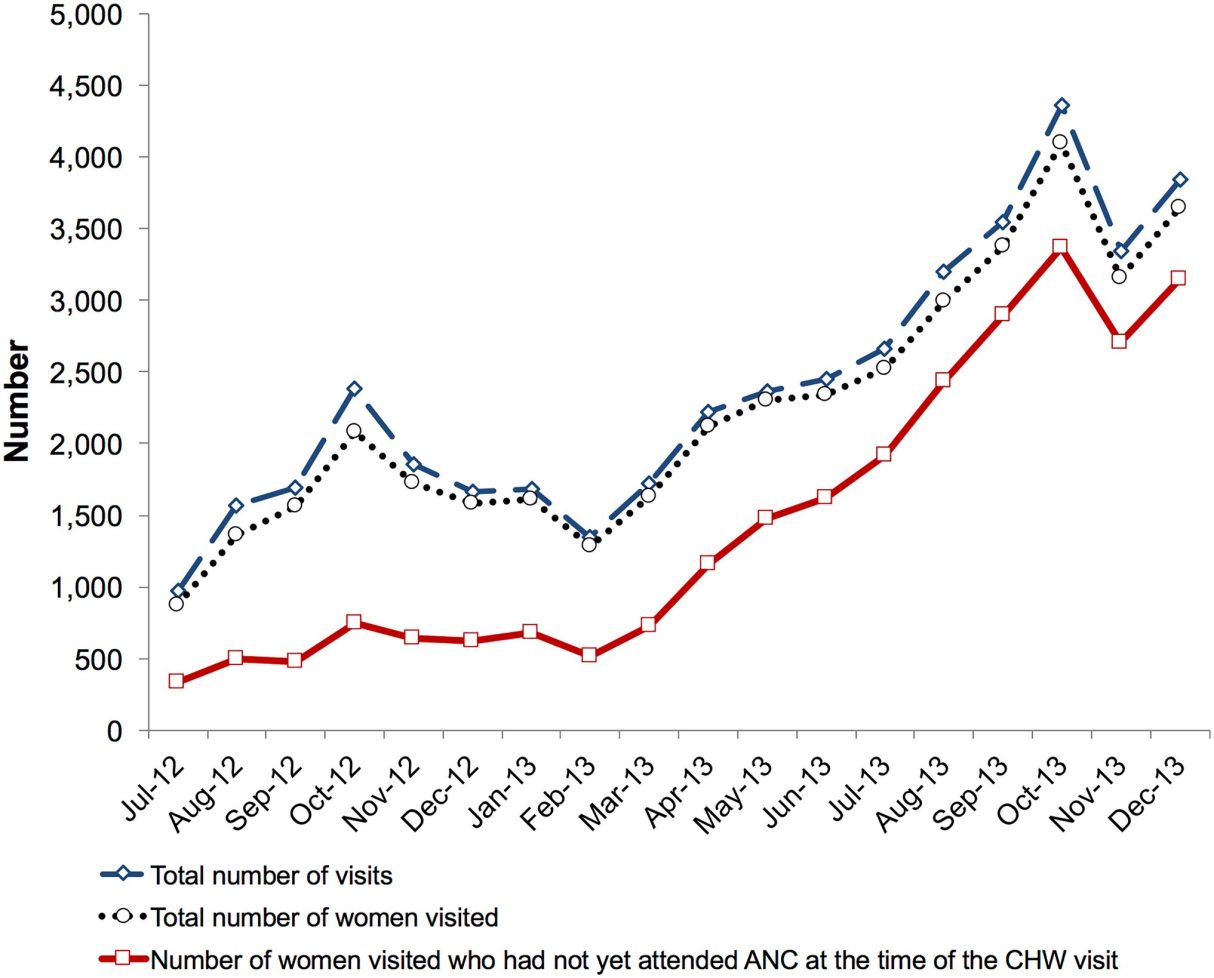
Home visits by CHWs over the study period, by month. These data were obtained from clinical registers that the CHWs in the intervention wards filled out and submitted to their supervising community outreach nurses at the healthcare facilities. The difference between the total number of visits and total number of women visited arose from the fact that some women were visited more than once during a given month. ANC, antenatal care; CHW, community health worker.

To elucidate the contextual conditions and mechanisms that might explain our trial results, we conducted a nested explanatory mixed-methods study. Analyzing quantitative data from the patient registers filled out by the CHWs in the intervention wards, we found that CHWs in the intervention wards reported having conducted a total of 45,216 visits to pregnant women over the study period, corresponding to a mean of 10.8 visits per CHW per month. The monthly number of visits, the number of women visited each month, and the proportion of these women who had not yet attended ANC at the time of the CHW visit all tended to increase over the study period (Fig 2). The median gestational age of a woman at the time of the first CHW visit decreased from 22 (IQR: 15–30) weeks in July 2012 to 17 (IQR: 12–23) weeks in January 2014 (S6 Table).

In the population-based household survey, 85.6% (95% CI: 81.4%–89.9%) of women in intervention wards reported that their household had ever been visited by a CHW—which could be a visit to the pregnant woman herself as part of the CHW intervention, or a visit to an HIV-positive patient (as part of regular HIV care in Dar es Salaam) in the household either during or prior to the study period—while only 37.4% (95% CI: 27.0%–47.9%) of women in the standard-of-care wards reported that their household had ever received a CHW visit. Among those who had ever been visited by a CHW, the mean and median number of CHW visits that respondents reported having received were the same (2.4 and 2.0 visits, respectively) among respondents in the intervention wards as among respondents in the standard-of-care wards.

While the qualitative interviews with pregnant women did not clarify why not all pregnant women and new mothers in the population-based survey were visited at least once, a common theme in the interviews with CHWs was that some household members were concerned that a CHW visit could signal ill health to neighbors. According to the CHWs, neighbors suspecting that a household member might be HIV-positive was a particular concern in this regard.

“For some houses, when you go there, the husband may be very harsh and tells you ‘you should never be seen by neighbors coming to my house again.’ And some women don’t want to be visited at home. They like to be called by phone and then arrange for a meeting with the [CHW] outside their home. They are afraid if, for example, people learn that they are HIV-infected.”—CHW

#### Was the quality of CHW visits insufficient?

Given that education and counseling was an important component of the CHW visits, Table 3 presents the answers to maternal health knowledge questions of participants in the population-based survey as evidence on the quality of the CHW visits. The proportion of women giving a wrong answer was lower in the intervention than the standard-of-care arm for 5 out of 6 knowledge questions, and almost the same between the arms for 1 knowledge question (Table 3). The differences for both questions about HIV—not knowing that pregnant women should be tested for HIV (RR: 0.31; 95% CI: 0.14–0.70) and not knowing that it is possible for a baby to be born with HIV (RR: 0.55; 95% CI: 0.31–0.95)—were statistically significant. For a further 2 questions, the differences had *p*-values between 0.05 and 0.1.

**Table 3:** Maternal health knowledge by study arm.

The respondent does *not* know that…	Intervention (%)	Standard of care (%)	Risk ratio (95% CI)	*p*-Value
one should deliver at a healthcare facility (*n =* 2,171)	2.9	5.5	0.53 (0.26–1.09)	0.083
one should attend ANC ≥4 times (*n =* 2,218)	19.2	27.0	0.71 (0.46–1.10)	0.128
one should attend ANC in the first trimester (*n =* 2,208)	29.1	28.7	1.01 (0.70–1.47)	0.943
pregnant women should get tested for HIV (*n =* 2,244)	2.8	9.0	0.31 (0.14–0.70)	0.005
it is possible for a baby to be born HIV-infected (*n =* 2,217)	9.5	17.3	0.55 (0.31–0.95)	0.031
exclusive breastfeeding is the recommended feeding method for the first 6 months after delivery (*n =* 2,243)	11.6	19.8	0.59 (0.33–1.05)	0.074

Standard errors were adjusted for clustering at the ward level.

ANC, antenatal care.

**Fig 3:**
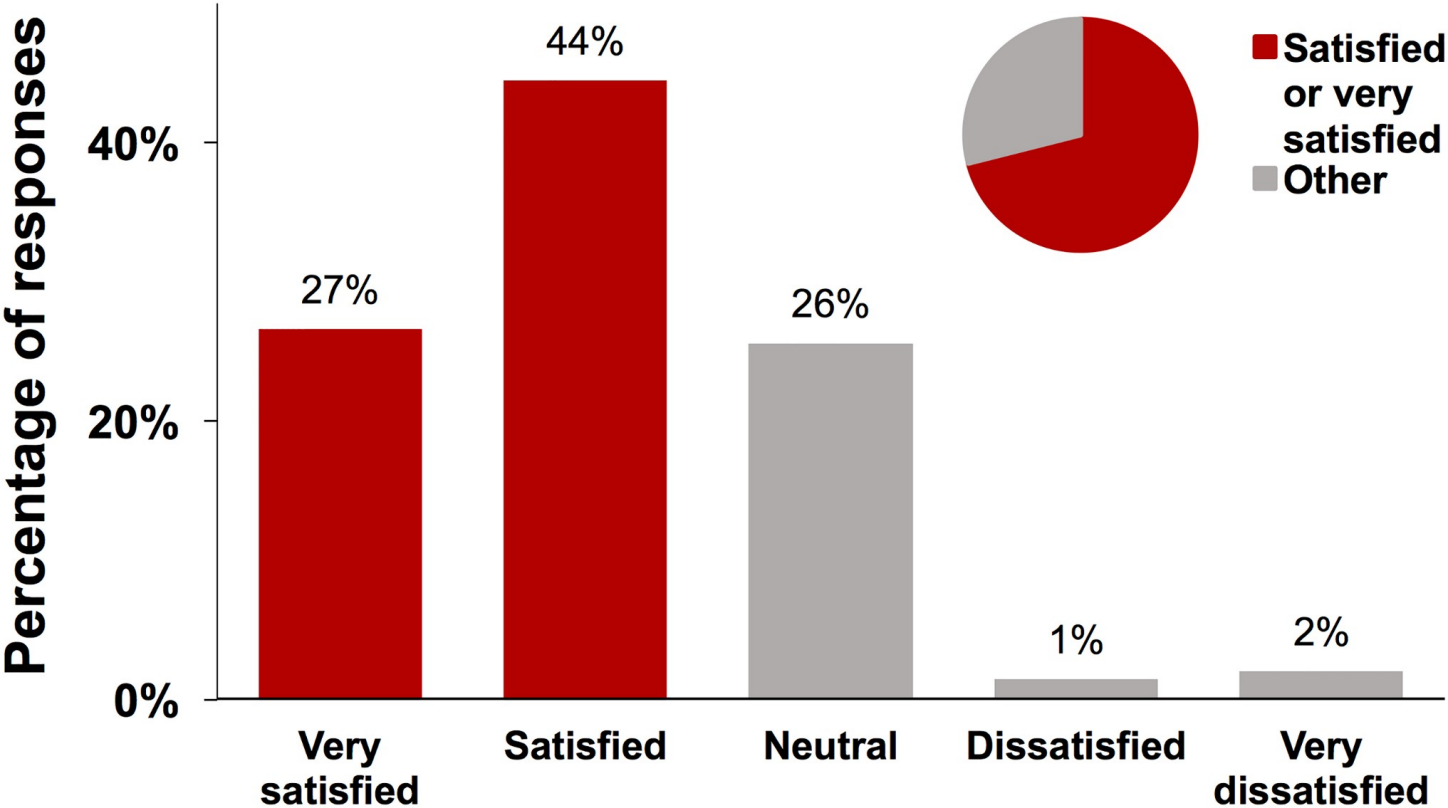
Distribution of responses to the question “How satisfied are you with the CHW program?”. In all, 1,354 participants answered this question.

Among the 1,431 women who reported having ever received a CHW visit, 71.1% stated being either “very satisfied” or “satisfied” with the CHW program (Fig 3); 25.6% said they were neutral, and 3.4% were either dissatisfied or very dissatisfied. The distribution was similar when restricting the sample to respondents in the intervention wards (S1 Fig). In qualitative interviews, several pregnant women and new mothers mentioned that the CHWs provided helpful and informative encouragement to attend ANC/PMTCT.

“[The CHW] told me to go to the clinic because these days they have many things to help sick people, including drugs that will help to protect the child…and using the drugs is easy, so I should go to the clinic.”—pregnant woman living with HIV

In addition, the pregnant women frequently expressed that the CHWs provided a high degree of companionship and emotional support during pregnancy and around delivery, which they appreciated.

“I am grateful that [the CHWs] were with me the whole way from pregnancy through birth and even now they call me.”—woman who delivered during the study period

“They really care for me and visit me; even if it is not a work day for that mama [CHW], she comes to my home.”—pregnant woman

In the qualitative interviews with CHWs and community outreach nurses, both cadres expressed a high degree of satisfaction with the training they had received for the intervention of this trial and felt that it had prepared them adequately for their new tasks.

“The trainings we received through the Familia Salama project have added a lot on improving our performance as healthcare workers and prepared us well.”—community outreach nurse

#### Did poor ANC quality discourage attendance?

**Fig 4:**
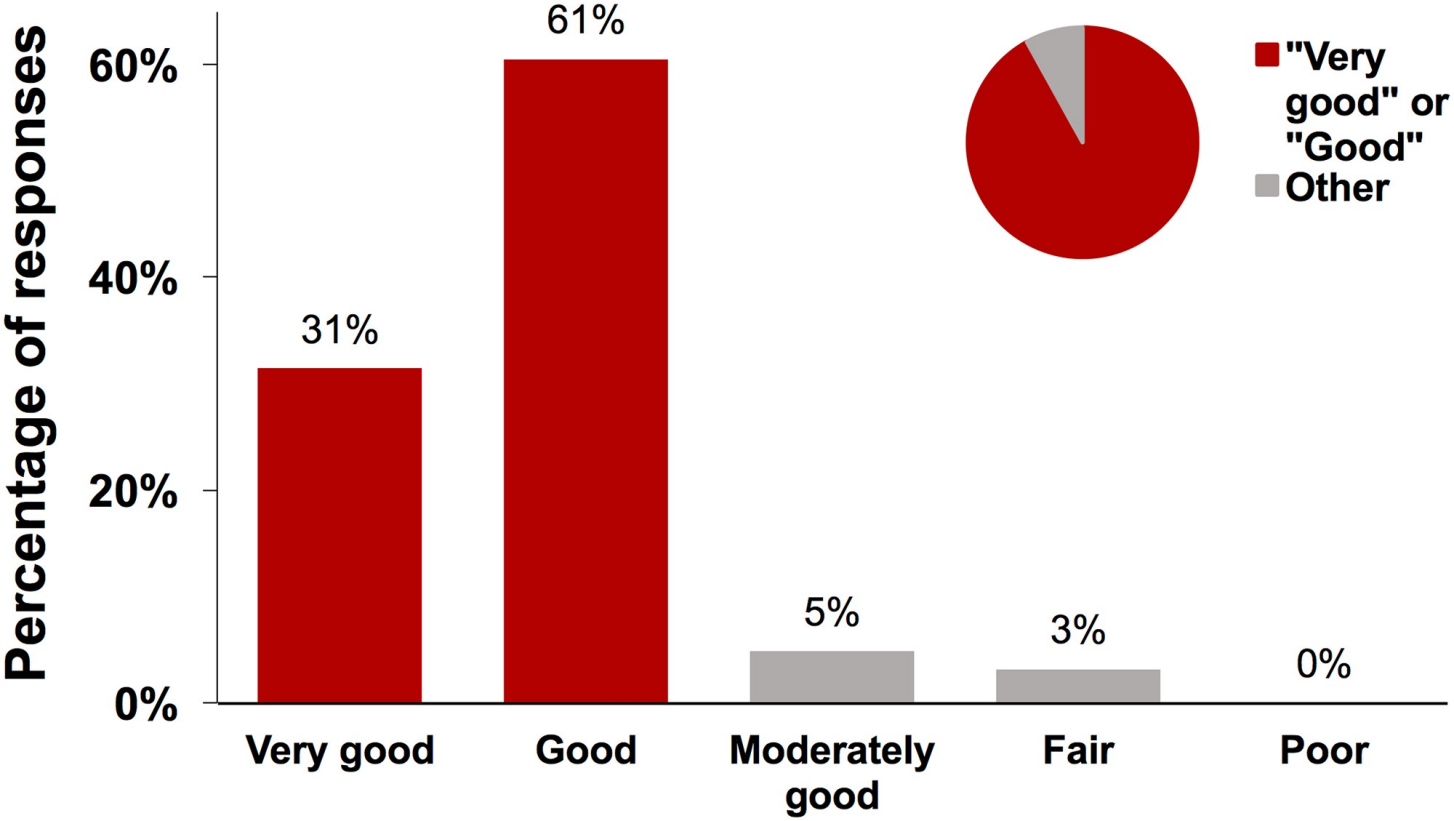
Distribution of responses to the question “How would you rate the overall quality of the healthcare that you received today?”. In all, 595 participants answered this question.

In the healthcare-facility-based patient satisfaction survey, 91.9% (95% CI: 88.9%–95.0%) of participants rated the overall quality of the healthcare that they received at their ANC or PMTCT visit as “very good” or “good” (Fig 4). No respondent rated the quality as “poor.” The distribution of responses was similar when participants were asked about each of the following domains of healthcare quality: (i) communication, (ii) confidentiality, (iii) promptness of attention, and (iv) perceived level of technical skills of the healthcare provider (S2–S5 Figs).

The qualitative interviews with pregnant women generally confirmed the impression from the patient satisfaction survey that ANC providers’ communication tended to be good.

“Yes, I will recommend ANC because the services are very good. First, when you go to ANC they welcome you in a very good way; they talk to you in a very polite way. In short, the services are good and the nurses are good listeners.”—pregnant woman

However, it is important to note that while the participants did not mention experiences of poor communication by healthcare providers during their own ANC visits, several nonetheless felt that disrespectful or unempathetic communication by the provider was a common deterrent to attending ANC for pregnant women in Dar es Salaam.

“Sometimes health workers talk about things in a way that they feel are normal but us pregnant women feel bad with the language used. They have to use language that will comfort the women.”—pregnant woman

Other domains of healthcare quality—apart from long waiting times, which is discussed in more detail below—were not mentioned by interviewees.

#### Did the economic burden or inconvenience of ANC discourage attendance?

**Fig 5:**
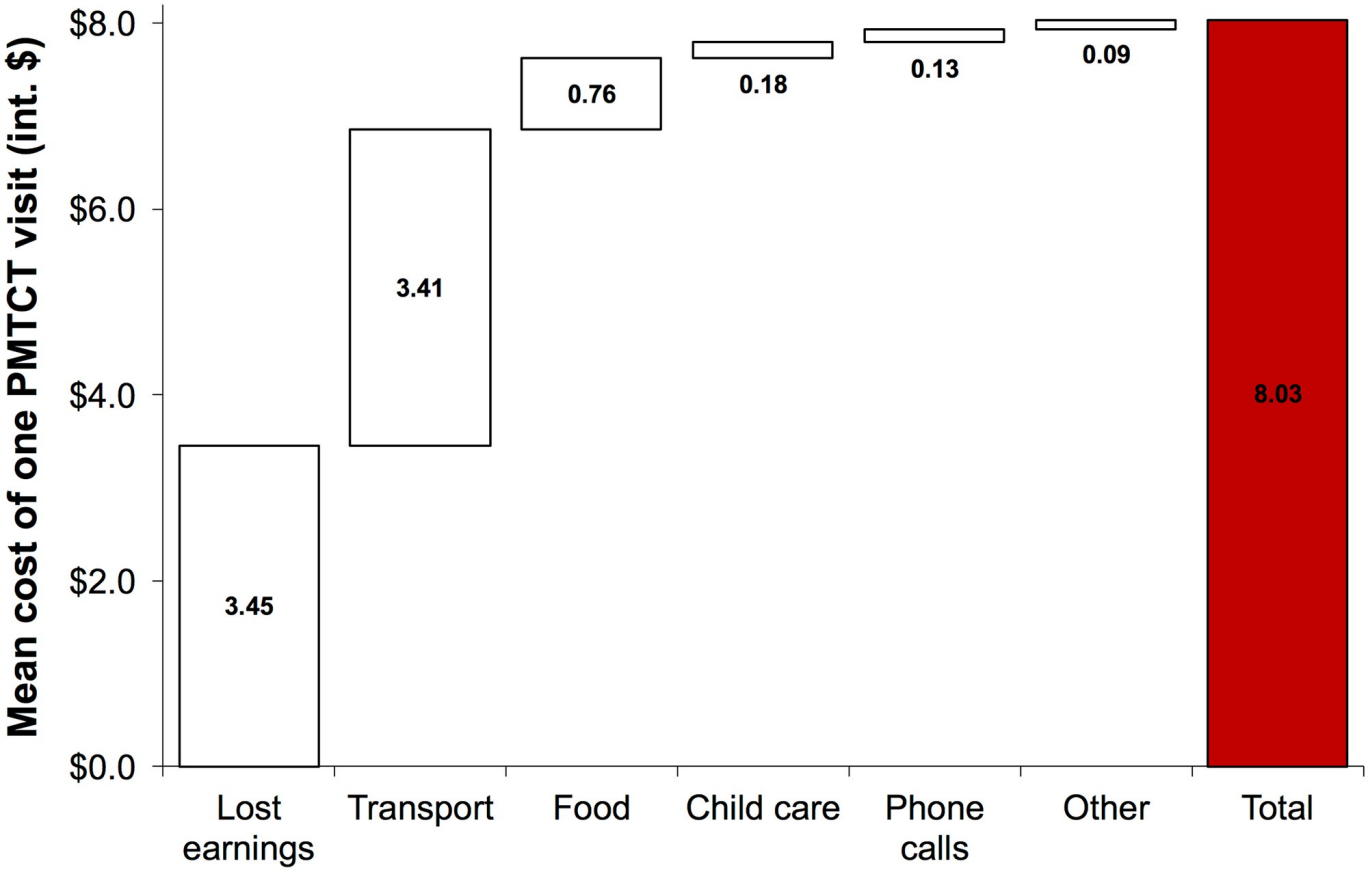
Mean cost to patients of attending 1 prevention of mother-to-child HIV transmission (PMTCT) visit. All monetary values were converted to purchasing power parity-adjusted international dollars (int$) [32]. These questions were answered by 595 participants.

Of participants in the healthcare-facility-based patient satisfaction survey, 28.0% (95% CI: 21.6%–34.5%) needed between 31 and 60 minutes to travel to the healthcare facility (1 way), and 20.5% (95% CI: 14.4%–26.6%) more than 1 hour. In all, 65.1% (95% CI: 53.5%–76.7%) of participants reported having spent at least 1 hour at the healthcare facility the last time they visited PMTCT; 23.0% (95% CI: 14.2%–31.9%) spent at least 3 hours. The mean cost of attending 1 PMTCT visit was int$8.03 (95% CI: int$6.27–int$9.79), with the median being int$2.99 (IQR: int$0.83–int$9.14). The largest costs for patients arose from lost income due to the time needed to attend the facility and from transport expenses (Fig 5).

In qualitative interviews, both pregnant women and CHWs noted that time spent attending the healthcare facility was an important barrier to attending ANC both earlier and more frequently:

“A pregnant woman might tell you for example ‘I would like to start clinic in July or August and give birth in September.’ You can see that she is looking for simplicity hoping to avoid many ANC clinic visits just because she doesn’t want to wait in the clinic for a long time to receive services.”—CHW

Interviewer: “What is your recommendation to increase ANC attendance?”

Participant: “To provide quick services. I mean, if you go there they should attend you without waiting for others to come. You may go to the ANC clinic early in the morning without taking breakfast but you will get services at 11 AM and stay there up to 1 PM. So, they have to improve on this.”—pregnant woman

Several CHWs felt that their provision of ANC referral cards to women as part of the CHW intervention may have reduced the waiting time at healthcare facilities for some women, although they did acknowledge that the referral cards were unlikely to affect waiting times at large healthcare facilities.

“The women like [the referral cards] especially if they are referred to small facilities as opposed to the large referral facilities with many people, because when they get there with such letters from us they are received and given first priority to get care and don’t wait for long. That has even made other women in the community seek out [CHW] services.”—CHW

None of the interviewed pregnant women, however, mentioned that the CHWs’ referral cards improved their waiting time. Some CHWs also acknowledged that their referral cards, which were for attendance of the nearest healthcare facility, may not have been used by HIV-positive pregnant women because they felt that many of these women prefer to attend care further away from their home to reduce the risk of accidental disclosure of their HIV status.

It is also possible that the intervention may have inadvertently increased waiting times at ANC facilities for those women who attended without a partner. The intervention encouraged partner involvement in ANC, which may have led to a higher probability of a woman attending ANC with her partner. Several women mentioned that those who attended ANC with their partner were seen faster than those without a partner, which was viewed as being unfair.

“There is such a thing of coming with your partner, whereby those who are coming with their partners are the ones who are getting first priority on services. Those who came alone have to wait regardless of their time of arriving at the clinic. Not every woman has a partner and they don’t deserve to receive services late simply because they have no partner.”—pregnant woman

## Discussion

In this cluster-randomized trial implemented directly in the public-sector health system in urban Tanzania, we investigated the impact of a large-scale community intervention—consisting of additional CHW supervision, CHW-led identification of newly pregnant women, and CHW follow-up at home of those who missed an ANC appointment—on ANC attendance and uptake of facility-based delivery. We found that the intervention had no effect on reducing self-reported late or infrequent ANC attendance. However, results suggest that the intervention was effective in reducing self-reported delivery at home. The CHW intervention almost halved the probability of self-reported home delivery, moving the already very high proportion of facility-based delivery in this population even closer to universal coverage.

We postulated 4 broad categories of reasons for why the CHW intervention was ineffective in affecting ANC outcomes: (i) the quantity of CHW visits was insufficient, (ii) the quality of CHW visits was insufficient, (iii) poor quality of ANC at healthcare facilities deterred people from attending, and (iv) the economic burden and inconvenience of ANC presented obstacles that were too large to overcome merely with information, encouragement, and reminders by CHWs. We examined these hypotheses using both quantitative and qualitative data for these implementation outcomes in a nested explanatory mixed-methods study. Regarding the first hypothesis, while not all (85.6% [95% CI: 81.4%–89.9%]) pregnant women in the intervention wards reported having been visited by a CHW, the exposure to CHWs appears to have been comparatively high and substantially greater than in the standard-of-care wards (37.4% [95% CI: 27.0%–47.9%]). Similarly, we found that the second reason—insufficient quality of CHW visits—was unlikely to be the main reason for the null effect on ANC outcomes. Specifically, an important component of the CHW visits was the provision of information and education on maternal health. For survey questions regarding maternal health knowledge, even though the absolute differences between the standard-of-care and intervention wards were all less than 10 percentage points and reached statistical significance for only 2 of the 6 knowledge questions, the CHW intervention appears to have had large general effects in reducing poor maternal health knowledge. However, it must be noted that—for reasons that are not clear to us—the largest relative effects of the intervention were observed for knowledge regarding facility-based delivery and PMTCT rather than ANC (with the question about ANC visit frequency having a non-significant effect favoring the intervention arm and no effect for the question on timeliness of the first ANC visit). In addition, most (71.1%) women rated their satisfaction with the CHW program as “very satisfied” or “satisfied.” In contrast, the economic burden and time needed to attend a PMTCT visit—and by extrapolation an ANC visit—seemed to have been substantial. In addition, the qualitative interviews with pregnant women indicated that the perceived economic burden and inconvenience of attending ANC was an important reason for attending late or infrequently. Thus, while our data on implementation outcomes can only be interpreted as being suggestive, it appears that merely providing information, encouragement, and reminders to attend ANC was insufficient to overcome women’s perceptions that the economic burden (particularly transport costs and time lost from income-generating activities) and inconvenience of ANC outweigh the possible benefits of attending ANC earlier and more frequently.

This finding generally agrees with those from qualitative studies on reasons for low use of ANC services in LMICs. For instance, in a meta-synthesis of 21 qualitative studies in LMICs, Finlayson and Downe identified 3 major themes for poor ANC uptake [33]. Two of these themes were (i) the economic burden (including fees, transport costs, and time lost from other responsibilities) and inconvenience (including time spent waiting at the healthcare facility) of attending ANC and (ii) the beliefs and preferences held by women regarding the necessity of ANC (including from which type of provider ANC services should be sought at what stage of pregnancy). The themes identified by this qualitative meta-synthesis were also reflected in the 2 studies from Tanzania—which were, however, both set in rural areas—included in the review [34,35]. One barrier to early ANC attendance that did not emerge from our data but may nonetheless have been important is reluctance to disclose a pregnancy during its early stages (e.g., because of shame due to pregnancy’s obvious connection to sexual activity, or to reduce the risk of being “cursed” by spirits or jealous peers), which was a theme identified by several studies included in the meta-synthesis [33].

A possible explanation for the beneficial effect we observed of the CHW intervention on facility-based delivery in the absence of an effect on the ANC outcomes is the phenomenon of time inconsistency, or present bias. This bias describes the observation that people tend to “procrastinate.” That is, they value gratification in the present over gratification in the future to a degree that exceeds a standard exponential discounting benchmark, and is therefore inconsistent with a rational model of utility maximization [36]. ANC attendance has a number of characteristics that make it more vulnerable to present bias than facility-based delivery: (i) the precise timing of ANC attendance during pregnancy is comparatively flexible, (ii) the benefit of ANC attendance may only be evident to a minority of ANC attendees (i.e., to women in whom the ANC clinician identifies a pregnancy complication), and (iii) the perceived marginal benefit of 1 further ANC visit (e.g., 4 versus 3 ANC visits during pregnancy) is likely to be low. Thus, despite the encouragement and counseling by CHWs, on any given day, many pregnant women were able to “put off” the immediate cost (e.g., time loss and transport costs) of attending ANC to a time in the near future without a substantial reduction in the perceived future rewards from attending ANC. In contrast, facility-based delivery is less vulnerable to present bias because women know that the decision of where to deliver cannot be substantially delayed once labor has begun. In addition, the benefits of delivering in a healthcare facility are likely to be more evident to women than those of attending ANC because delivery is probably perceived as posing a greater risk to the health of both the mother and the newborn than pregnancy [37].

CHW interventions in LMICs to improve uptake of ANC and facility-based delivery have generally shown encouraging results, although the evidence base from large-scale trials implemented in the public-sector health system is sparse [16,17,19,20]. While it is difficult to compare our findings to those of other studies given the large degree of variation in the characteristics of CHW cadres and differences in intervention designs, local health system contexts, and outcome assessments, we believe that the reduction in home delivery observed in this trial should be appreciated in light of the already very high uptake of facility-based delivery (92.7% in standard-of-care wards) in Dar es Salaam. In our view, increasing the utilization of facility-based delivery in Dar es Salaam may well constitute a “last mile” health problem. Thus, unlike for the ANC outcomes, it is plausible that the CHW intervention would have resulted in a larger effect size for improving uptake of facility-based delivery than observed in this trial if it had been implemented in a setting with a lower uptake of facility-based delivery at baseline.

Other generalizability considerations of this trial include that it took place in a highly urbanized setting with a high density of healthcare facilities and ample availability of transport to reach a variety of facilities. Our results are thus unlikely to apply to rural settings in sub-Saharan Africa. Similarly, CHW programs tend to vary between countries in their training, supervision structure, and remuneration [16,38]. Nonetheless, the CHWs who implemented most of the components of the intervention received a minimal amount of pre-service training, needed to have completed only primary school education, earned a small stipend compared to local living expenses, and mostly worked only part time. On the assumption that more highly trained, better educated, and better-paid CHWs would not deliver a lower quantity and quality of services than the CHWs in this trial, the effect sizes observed in this study could be viewed as a minimum. It is, thus, plausible that the same intervention delivered by a different CHW program in a different setting may have achieved a beneficial effect on ANC attendance and a larger positive effect than that identified in this study on facility-based delivery.

This study has several limitations in addition to generalizability considerations. First, the outcomes were assessed through self-report only, which is a potential source of bias that may have been increased by the fact that CHWs acted as data collectors for the population-based household survey. Specifically, women may have wanted to please the CHWs through reporting the behavior that CHWs recommended during their visits, and this bias would likely be stronger among women who were visited by CHWs during the study period. To reduce this bias, CHWs conducted the household survey in neighborhoods in which they did not work as a CHW. Respondents were thus not familiar with the CHW who visited them during the household survey. Nonetheless, CHWs may also have had an incentive to make the intervention appear as if it had a positive impact in order to please a supervisor or for their own personal job satisfaction. This may have resulted, whether consciously or subconsciously, in CHWs asking questions (or even reporting answers) differently between women who reported having been visited by a CHW and those who did not, or between women in the standard-of-care and intervention wards. The fact that the data show no beneficial effect of the intervention on any of the ANC outcomes suggests, however, that the use of CHWs as data collectors for the household survey may not have been a major source of bias in this study.

Second, we were unable to assess whether the increased CHW focus on maternal healthcare in the intervention arm had any negative consequences on CHWs’ usual responsibilities of visiting HIV patients. As this intervention added supervision and support to CHWs through additional community outreach nurses, it could have had both a positive and a negative effect on the routine areas of CHW work.

Third, the response rate was not 100%, and differed somewhat between the intervention and standard-of-care wards (93.3% [1,664/1,784] versus 87.7% [665/758], respectively). If women who could not be reached for the household survey were systematically less likely than survey participants to report having delivered at a healthcare facility and/or having visited ANC at the recommended time and frequency, then our results would underestimate the benefit of the CHW intervention for these outcomes. The opposite would be true if women who were not reached for the survey were *more* likely to report having delivered at a healthcare facility or to have attended recommended ANC than women included in the survey. In addition, there was a moderate degree of missing observations for the outcome variables. While the percentage missing was similar between study arms, the missing data would still bias our results if those with a missing value had systematically different outcomes from those included in the analysis.

Fourth, one data source in which we examined 2 possible barriers to earlier and more frequent ANC attendance—poor quality of care and the economic burden and inconvenience of attending ANC—was exit interviews among PMTCT patients at healthcare facilities. A limitation of this approach is that the perceived quality of care and/or economic burden and inconvenience of attending care among this population of pregnant women, who are living with HIV and attending care, may not be generalizable to all pregnant women in the study area.

Lastly, the trial was ended 4 months earlier than planned. Thus, if the healthcare workers implementing the intervention delivered an increasing quantity and/or quality of intervention activities per unit of time as the study period progressed (as is suggested by the increasing number of CHW home visits over time shown in Fig 2)—for example, because they learned over time how to carry out the activities more efficiently—then it is possible that the results would have shown larger beneficial effects of the intervention if the study had lasted for a longer duration.

This cluster-randomized pragmatic trial, implemented in 2 out of the 3 districts in Dar es Salaam, evaluated the impact of an intervention consisting of additional CHW responsibilities for maternal health and additional CHW supervision by community outreach nurses. The CHW intervention appears to have been highly effective in reducing home delivery. However, the intervention did not affect ANC uptake, probably because the information, encouragement, and reminders provided by the CHWs were insufficient to overcome women’s perception that the benefits of ANC do not outweigh the associated economic burden and inconvenience. Future studies should investigate the impact of models of care delivery that reduce the cost and inconvenience of receiving ANC—such as the shifting of essential ANC tasks from facility-based nurses to CHWs—on maternal care uptake, and on health outcomes of the mother and the newborn.

## Supporting information

S1 Fig Distribution of responses to the question “How satisfied are you with the CHW program?” among participants in intervention wards. In total, 1,177 participants answered this question.
(TIF)

S2 Fig Distribution of responses to the question “Overall, how would you rate your experience of how well healthcare providers communicated with you during this visit?”. In total, 586 participants answered this question.
(TIF)

S3 Fig Distribution of responses to the question “Overall, how would you rate your experience of the way the clinic kept information about you confidential?”. In total, 587 participants answered this question.
(TIF)

S4 Fig Distribution of responses to the question “Overall, how would you rate the promptness of attention at this clinic today?”. In total, 589 participants answered this question.
(TIF)

S5 Fig Distribution of responses to the question “How were the technical skills of healthcare providers?”. In total, 589 participants answered this question.
(TIF)

S1 Table Sample characteristics of participants in the patient satisfaction survey. (DOCX)

S2 Table ANC attendance outcomes by study arm, restricted to women who had completed 9 months of pregnancy. (DOCX)

S3 Table Place of delivery and ANC attendance by study arm, adjusted for age group and education. (DOCX)

S4 Table Place of delivery and ANC attendance by study arm when excluding women for whom pregnancy status (currently pregnant or recently delivered) was unclear. (DOCX)

S5 Table Place of delivery and ANC attendance by study arm, using logistic instead of log-binomial regression. (DOCX)

S6 Table Quantity of home visits conducted by CHWs over the study period. (DOCX)

S1 Text Questionnaire for the population-based survey. (DOCX)

S2 Text Patient satisfaction questionnaire. (DOCX)

S1 Checklist CONSORT checklist. (DOCX)

## Abbreviations

ANC: antenatal care
ART: antiretroviral therapy
CHW: community health worker
ICC: intracluster correlation coefficient
int$: international dollars
LMICs: low- and middle-income countries
PMTCT: prevention of mother-to-child HIV transmission
RR: risk ratio
WHO: World Health Organization
