# Supplementary figures and images for: Community health workers to improve uptake of maternal healthcare services: A cluster-randomized pragmatic trial in Dar es Salaam, Tanzania
Source: PLoS Med. 2019 Mar 29;16(3):e1002768. doi: 10.1371/journal.pmed.1002768 (PMC6440613; doi:10.1371/journal.pmed.1002768)

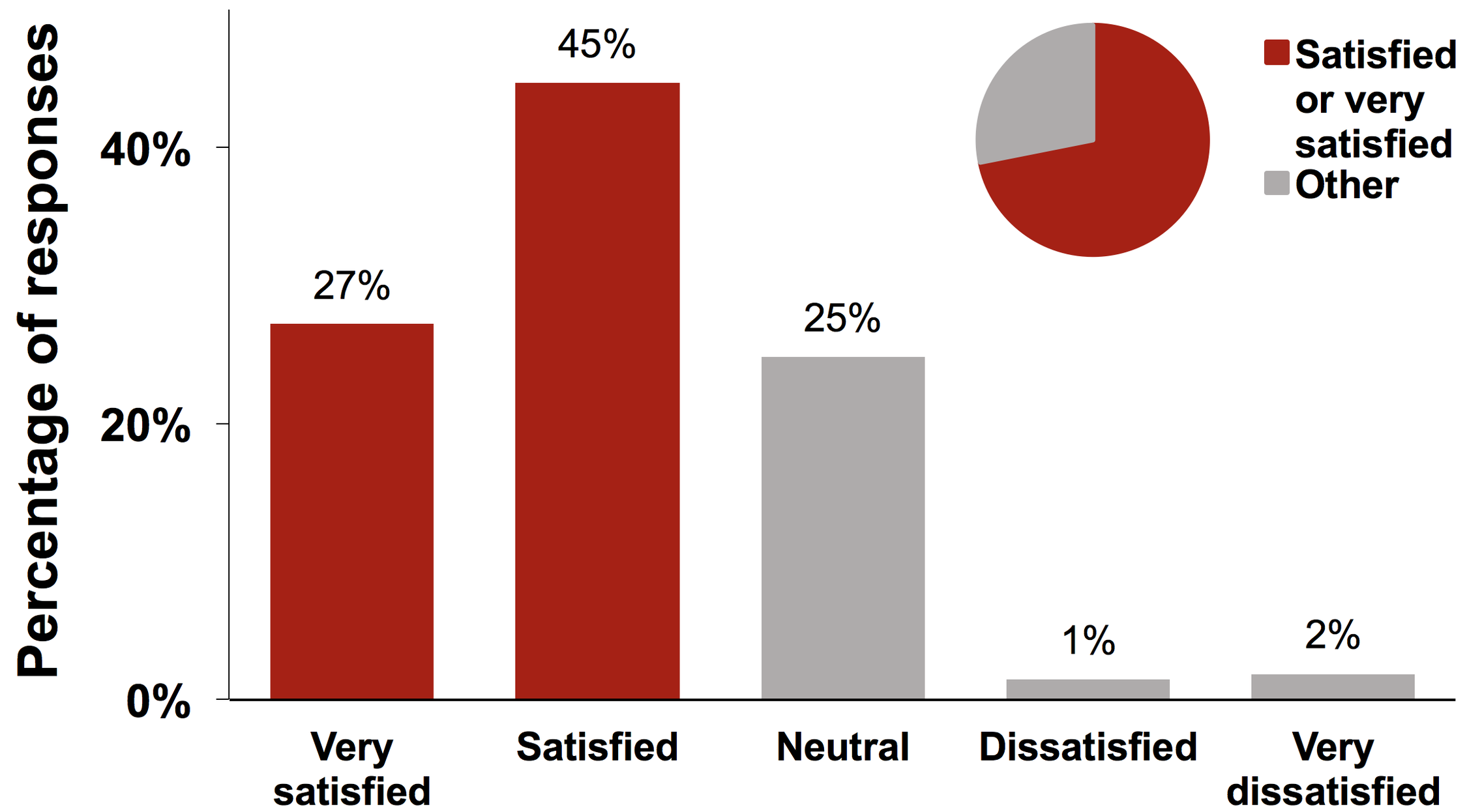

Supplement: S1 Fig — In total, 1,177 participants answered this question. (TIF) [file pmed.1002768.s001.tif]

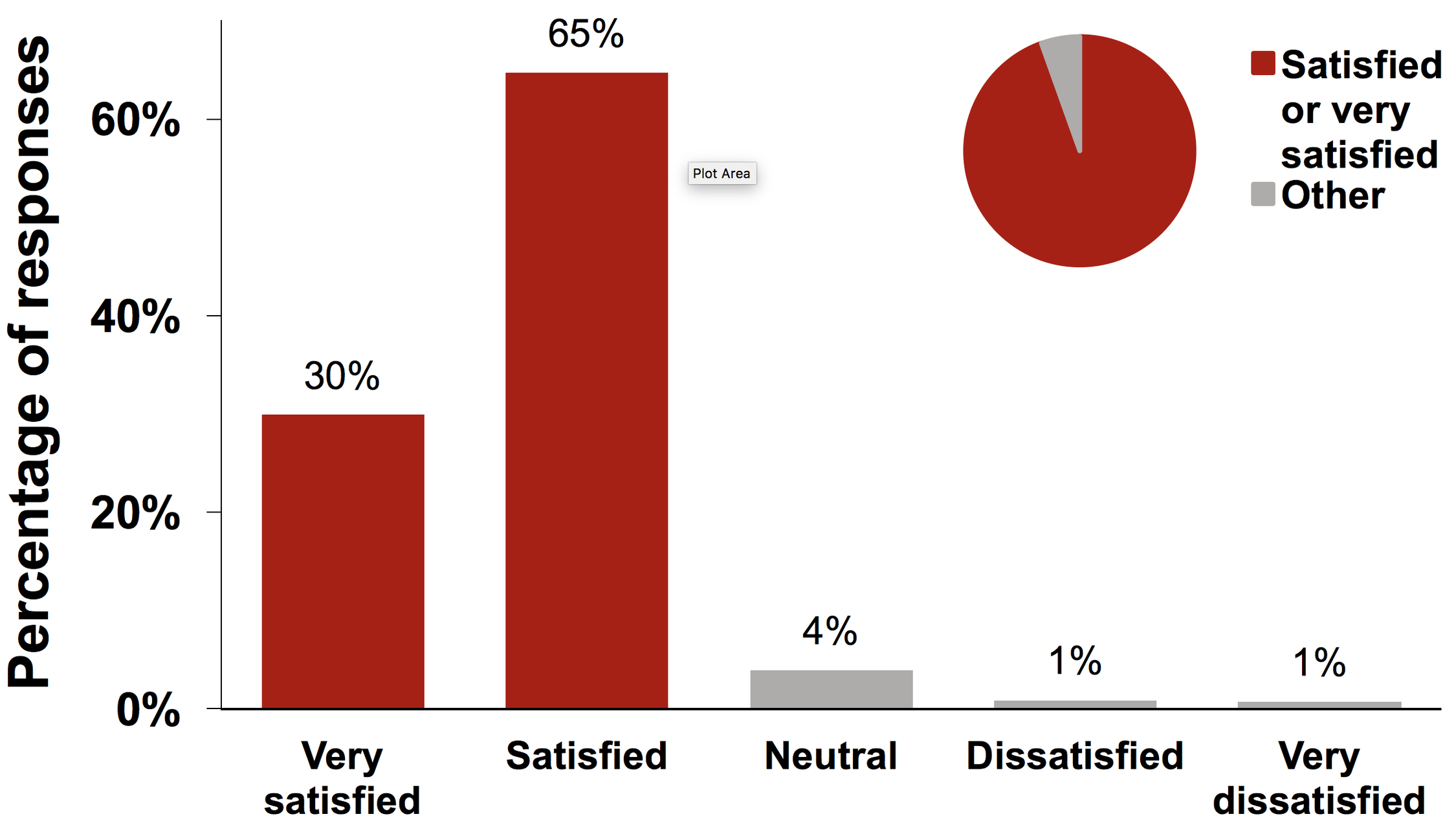

Supplement: S2 Fig — In total, 586 participants answered this question. (TIF) [file pmed.1002768.s002.tif]

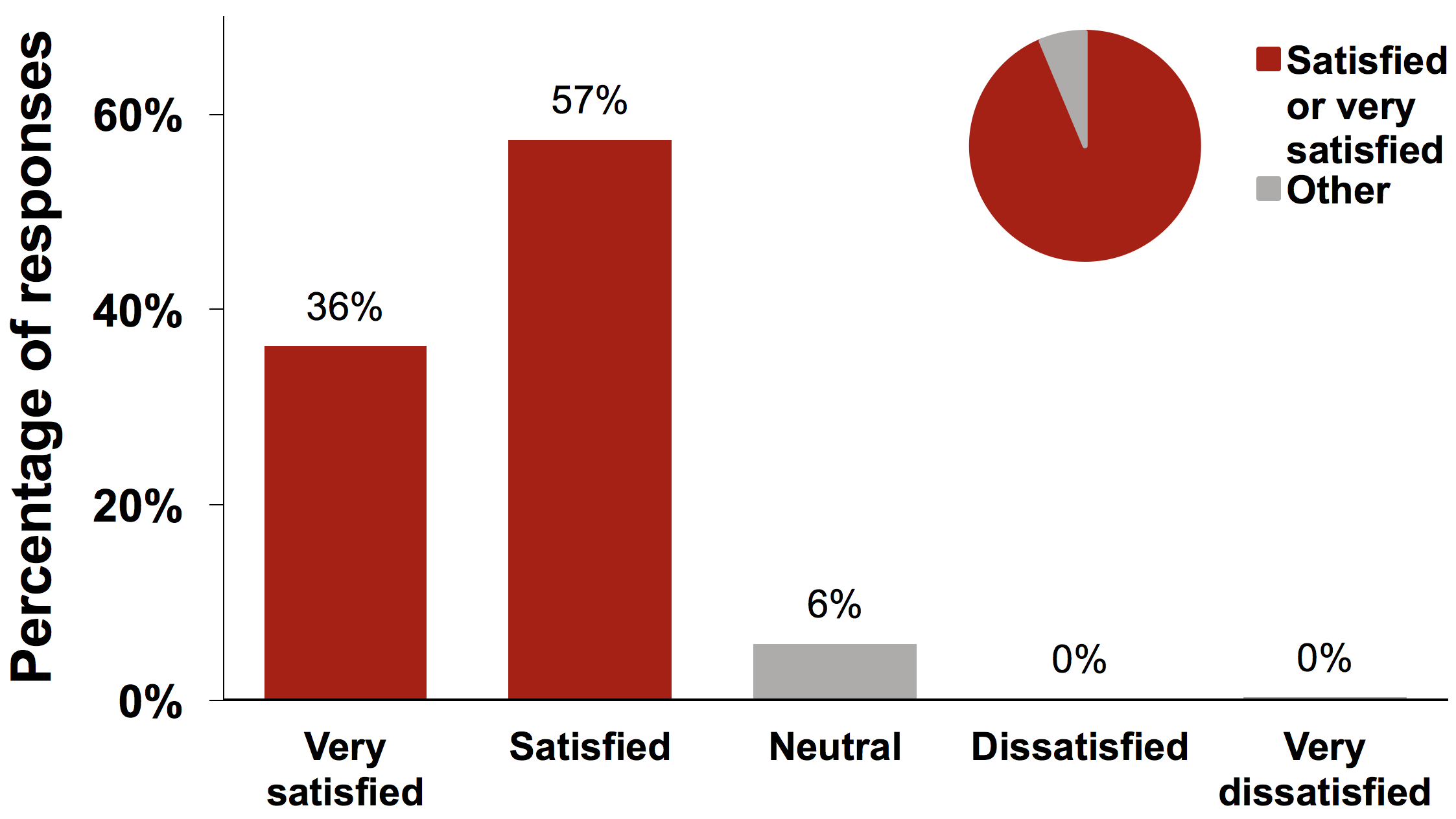

Supplement: S3 Fig — In total, 587 participants answered this question. (TIF) [file pmed.1002768.s003.tif]

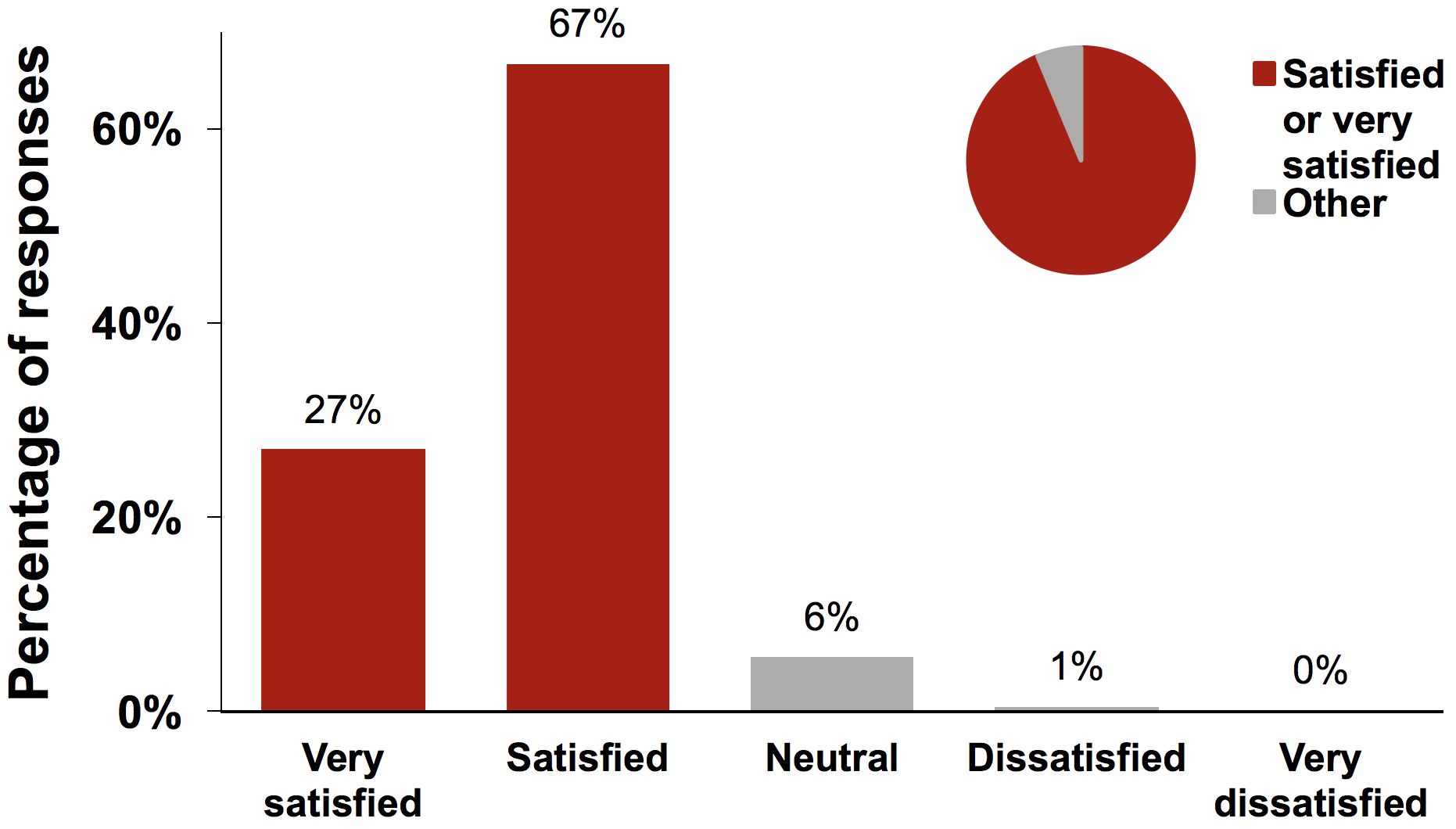

Supplement: S4 Fig — In total, 589 participants answered this question. (TIF) [file pmed.1002768.s004.tif]

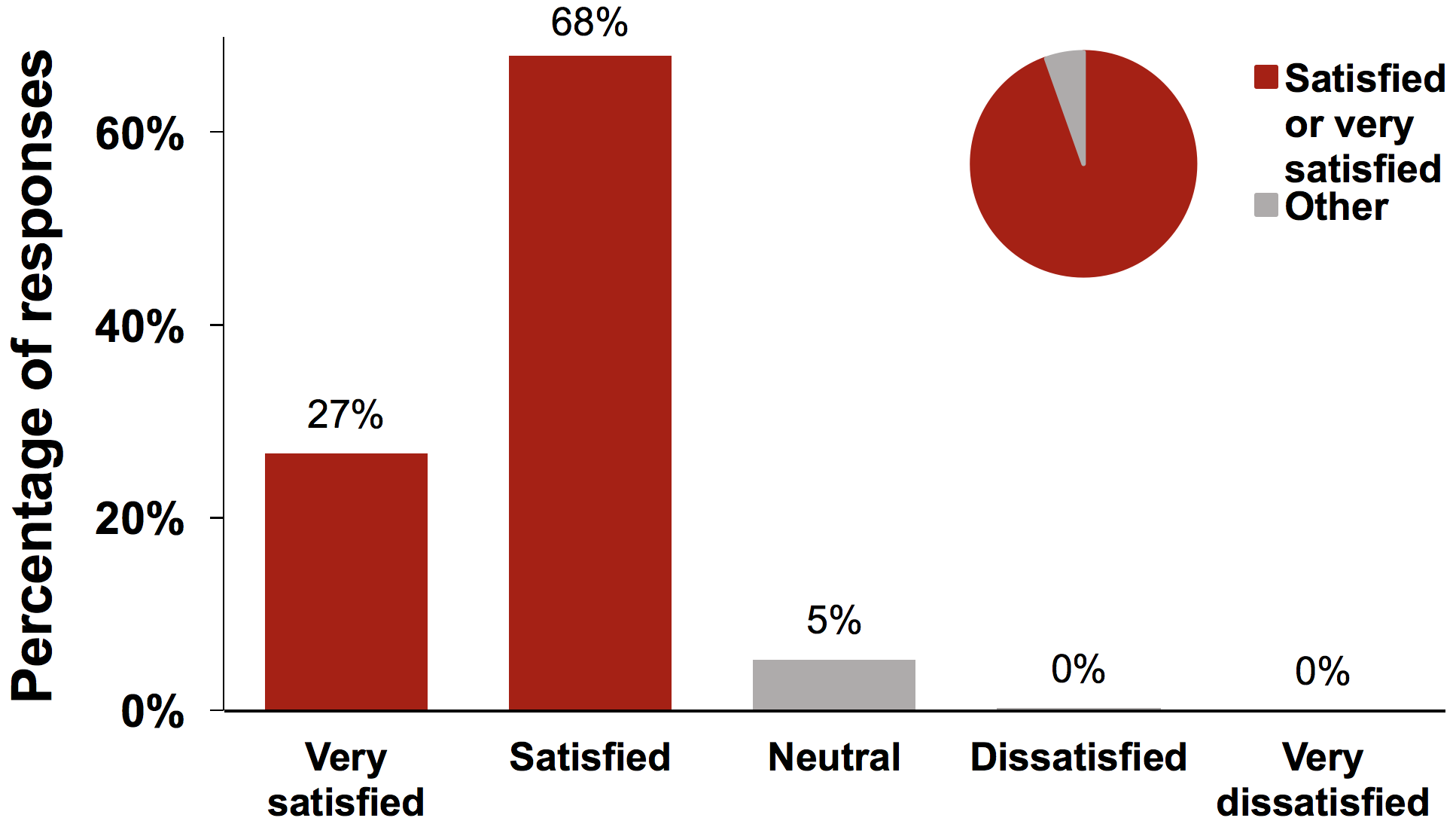

Supplement: S5 Fig — In total, 589 participants answered this question. (TIF) [file pmed.1002768.s005.tif]
