# Supplementary material for: Community health workers to improve uptake of maternal healthcare services: A cluster-randomized pragmatic trial in Dar es Salaam, Tanzania
Source: PLoS Med. 2019 Mar 29;16(3):e1002768. doi: 10.1371/journal.pmed.1002768 (PMC6440613; doi:10.1371/journal.pmed.1002768)
Supplement: S1 Table — (DOCX) [file pmed.1002768.s006.docx]

**Table S1. Sample characteristics of participants in the patient satisfaction survey**

| **Characteristic** | **Statistic** |
| --- | --- |
| N | 595 |
| Age, mean (SD) | 30.2 (5.3) |
| Age group, n (%) |  |
| 17-19 | 10 (1.8%) |
| 20-24 | 69 (12.7%) |
| 25-29 | 167 (30.6%) |
| 30-34 | 186 (34.1%) |
| 35-39 | 89 (16.3%) |
| ≥40 | 24 (4.4%) |
| Number of births, n (%) |  |
| 0 | 14 (2.7%) |
| 1 | 132 (25.2%) |
| 2 | 186 (35.5%) |
| 3 | 118 (22.5%) |
| ≥4 | 74 (14.1%) |
| Married, n (%) | 320 (55.1%) |
| Education, n (%) |  |
| Not been to school | 60 (10.1%) |
| Primary Education | 421 (70.8%) |
| Secondary Education (form I-IV) | 99 (16.6%) |
| Secondary Education (form V-VI) | 15 (2.5%) |
| Current occupation, n (%) |  |
| Unemployed | 123 (21.0%) |
| Employed | 65 (11.1%) |
| Self-employed | 187 (31.9%) |
| Housewife | 211 (36.0%) |
| Monthly household expenditure, n (%) |  |
| <50,000 TSh | 19 (3.5%) |
| 50,000 – 100,000 TSh | 134 (24.6%) |
| 101,000 - 300,000 TSh | 288 (52.8%) |
| 301,000 - 500,000 TSh | 87 (16.0%) |
| >500,000 TSh | 17 (3.1%) |

Abbreviations: SD=Standard deviation; TSh=Tanzanian Shilling
