# Supplementary material for: Community health workers to improve uptake of maternal healthcare services: A cluster-randomized pragmatic trial in Dar es Salaam, Tanzania
Source: PLoS Med. 2019 Mar 29;16(3):e1002768. doi: 10.1371/journal.pmed.1002768 (PMC6440613; doi:10.1371/journal.pmed.1002768)
Supplement: S2 Table — (DOCX) [file pmed.1002768.s007.docx]

**Table S2. ANC attendance outcomes by study arm, restricted to women who had completed nine months of pregnancy^1^**

|  | **Intervention**  (%) | **Control**  (%) | **Risk Ratio (95% CI)** | **P-value** |
| --- | --- | --- | --- | --- |
| Attended ANC < 4 times (n= 1,280) | 52.7 | 49.4 | 1.07 (0.84 – 1.35) | 0.590 |
| Did not attend ANC in first trimester (n=1,418) | 69.7 | 72.9 | 0.96 (0.87 – 1.05) | 0.366 |
| Never attended ANC (n= 1,486) | 3.3 | 3.9 | 0.85 (0.34 – 2.16) | 0.740 |

Abbreviations: ANC=antenatal care; CI=confidence interval

^1^ Standard errors were adjusted for clustering at the ward level.
