# Supplementary material for: Community health workers to improve uptake of maternal healthcare services: A cluster-randomized pragmatic trial in Dar es Salaam, Tanzania
Source: PLoS Med. 2019 Mar 29;16(3):e1002768. doi: 10.1371/journal.pmed.1002768 (PMC6440613; doi:10.1371/journal.pmed.1002768)
Supplement: S3 Table — (DOCX) [file pmed.1002768.s008.docx]

**Table S3. Place of delivery and ANC attendance by study arm, adjusted for age group and education^1,2^**

|  | **Risk Ratio (95% CI)** | **P-value** |
| --- | --- | --- |
| **Delivery** |  |  |
| Delivered at home^3^ (n=1,252) | 0.57 (0.32 – 1.01) | 0.055 |
| Does **not** intend to deliver in a healthcare facility^4^ (n=717) | 0.30 (0.10 – 0.93) | 0.037 |
| **ANC attendance^5^** |  |  |
| Attended ANC < 4 times (n=1,801) | 0.97 (0.83 – 1.15) | 0.746 |
| Did not attend ANC in first trimester (n=1,941) | 0.99 (0.88 – 1.12) | 0.904 |
| Never attended ANC (n=2,067) | 0.80 (0.37 – 1.76) | 0.582 |

Abbreviations: ANC=antenatal care; CI=confidence interval

^1^ Standard errors were adjusted for clustering at the ward level.

^2^ All models were adjusted for five-year age group and a binary indicator for having completed secondary school or a tertiary education.

^3^ This question was asked only to women who delivered within the previous two years.

^4^ This question was asked only to currently pregnant women.

^5^ During the current pregnancy (for currently pregnant women) or the most recent pregnancy (for women who delivered within the previous two years).
