# Supplementary material for: Community health workers to improve uptake of maternal healthcare services: A cluster-randomized pragmatic trial in Dar es Salaam, Tanzania
Source: PLoS Med. 2019 Mar 29;16(3):e1002768. doi: 10.1371/journal.pmed.1002768 (PMC6440613; doi:10.1371/journal.pmed.1002768)
Supplement: S4 Table — (DOCX) [file pmed.1002768.s009.docx]

**Table S4. Place of delivery and ANC attendance by study arm when excluding women for whom pregnancy status (currently pregnant or recently delivered) was unclear^1^**

|  | **Intervention**  (%) | **Control**  (%) | **Risk Ratio (95% CI)** | **P-value** |
| --- | --- | --- | --- | --- |
| **Delivery** |  |  |  |  |
| Delivered at home^2^ (n=1,277) | 3.8 | 6.6 | 0.57 (0.31 – 1.05) | 0.073 |
| Does **not** intend to deliver in a healthcare facility^3^ (n=739) | 1.2 | 3.1 | 0.38 (0.12 – 1.25) | 0.112 |
| **ANC attendance^4^** |  |  |  |  |
| Attended ANC < 4 times (n= 1,855) | 58.4 | 60.0 | 0.97 (0.81 – 1.16) | 0.767 |
| Did not attend ANC in first trimester (n=1,994) | 70.2 | 70.8 | 0.99 (0.87 – 1.12) | 0.893 |
| Never attended ANC (n= 2,121) | 4.9 | 6.2 | 0.78 (0.35 – 1.78) | 0.560 |

Abbreviations: ANC=antenatal care; CI=confidence interval

^1^ Standard errors were adjusted for clustering at the ward level.

^2^ This question was asked only to women who delivered within the previous two years.

^3^ This question was asked only to currently pregnant women.

^4^ During the current pregnancy (for currently pregnant women) or the most recent pregnancy (for women who delivered within the previous two years).
