# Supplementary material for: Community health workers to improve uptake of maternal healthcare services: A cluster-randomized pragmatic trial in Dar es Salaam, Tanzania
Source: PLoS Med. 2019 Mar 29;16(3):e1002768. doi: 10.1371/journal.pmed.1002768 (PMC6440613; doi:10.1371/journal.pmed.1002768)
Supplement: S5 Table — (DOCX) [file pmed.1002768.s010.docx]

**Table S5. Place of delivery and ANC attendance by study arm, using logistic instead of log-binomial regression^1^**

|  | **Odds Ratio (95% CI)** | **P-value** |
| --- | --- | --- |
| **Delivery** |  |  |
| Delivered at home^2^ (n=1,374) | 0.52 (0.28 – 0.95) | 0.035 |
| Does **not** intend to deliver in a healthcare facility^3^ (n=800) | 0.34 (0.12 – 0.94) | 0.038 |
| **ANC attendance^4^** |  |  |
| Attended ANC < 4 times (n= 1,979) | 0.94 (0.61 – 1.43) | 0.755 |
| Did not attend ANC in first trimester (n=2,135) | 0.98 (0.64 – 1.49) | 0.910 |
| Never attended ANC (n= 2,279) | 0.80 (0.64 – 1.49) | 0.605 |

Abbreviations: ANC=antenatal care; CI=confidence interval

^1^ Standard errors were adjusted for clustering at the ward level.

^2^ This question was asked only to women who delivered within the previous two years.

^3^ This question was asked only to currently pregnant women.

^4^ During the current pregnancy (for currently pregnant women) or the most recent pregnancy (for women who delivered within the previous two years).
