# Supplementary material for: Community health workers to improve uptake of maternal healthcare services: A cluster-randomized pragmatic trial in Dar es Salaam, Tanzania
Source: PLoS Med. 2019 Mar 29;16(3):e1002768. doi: 10.1371/journal.pmed.1002768 (PMC6440613; doi:10.1371/journal.pmed.1002768)
Supplement: S6 Table — (DOCX) [file pmed.1002768.s011.docx]

| **Month** | **Total no. of visits** | **Total no. of women visited** | **Women visited who had not yet attended ANC, n (%)^2^** | **Median gestational age at first CHW visit, weeks (IQR)** |
| --- | --- | --- | --- | --- |
| July 2012 | 970 | 869 | 337 (38.8%) | 22 (15, 30) |
| August 2012 | 1,572 | 1,357 | 496 (36.6%) | 22 (15, 29) |
| September 2012 | 1,690 | 1,559 | 482 (30.9%) | 23 (16, 30) |
| October 2012 | 2,379 | 2,078 | 747 (35.9%) | 21 (15, 28) |
| November 2012 | 1,859 | 1,723 | 637 (37.0%) | 21 (16, 27) |
| December 2012 | 1,667 | 1,586 | 623 (39.3%) | 22 (16, 28) |
| January 2013 | 1,683 | 1,606 | 675 (42.0%) | 21 (16, 29) |
| February 2013 | 1,349 | 1,287 | 517 (40.2%) | 22 (15, 29) |
| March 2013 | 1,719 | 1,631 | 730 (44.8%) | 21 (14, 29) |
| April 2013 | 2,223 | 2,117 | 1,162 (54.9%) | 19 (14, 26) |
| May 2013 | 2,365 | 2,297 | 1,474 (64.2%) | 18 (13, 24) |
| June 2013 | 2,455 | 2,342 | 1,619 (69.1%) | 18 (13, 23) |
| July 2013 | 2,663 | 2,524 | 1,914 (75.8%) | 18 (12, 23) |
| August 2013 | 3,201 | 2,991 | 2,439 (81.5%) | 16 (12, 21) |
| September 2013 | 3,549 | 3,373 | 2,899 (85.9%) | 16 (12, 21) |
| October 2013 | 4,356 | 4,098 | 3,368 (82.2%) | 16 (12, 21) |
| November 2013 | 3,343 | 3,154 | 2,709 (85.9%) | 16 (12, 21) |
| December 2013 | 3,844 | 3,641 | 3,149 (86.5%) | 17 (12, 22) |
| January 2014^3^ | 2,329 | 2,190 | 1,799 (82.1%) | 17 (12, 23) |

**Table S6. Quantity of home visits conducted by community health workers over the study period^1^**

**Abbreviations:** no.=number; ANC=antenatal care; CHW=community health worker; IQR=interquartile range

^1^ These data were obtained from clinical registers that the CHWs in the intervention areas filled out and submitted to their supervisors (the community outreach nurses) at the healthcare facilities.

^2^ The denominator for this percentage is the total number of women visited in the given month.

^3^ For this month we only had data for the first two weeks because the trial stopped on January 15^th^ 2014.
