# Supplementary material for: Community health workers to improve uptake of maternal healthcare services: A cluster-randomized pragmatic trial in Dar es Salaam, Tanzania
Source: PLoS Med. 2019 Mar 29;16(3):e1002768. doi: 10.1371/journal.pmed.1002768 (PMC6440613; doi:10.1371/journal.pmed.1002768)
Supplement: S1 Text — (DOCX) [file pmed.1002768.s012.docx]

**Text S1. Questionnaire for the population-based survey**

Management and Development for Health (MDH) is currently carrying out the Population Based project. As part of this project, we would like to find out the community’s rate of pregnancies, satisfaction with the Home-Based Carer (HBC) program, knowledge of maternal health issues and nutrition. Individual questionnaires will only be seen by MDH’s data management team and not passed to any outsiders. It should take approximately 45 minutes to complete the questionnaire.

**Instructions to HBCs:**

*Please introduce yourself and state the purpose of the study, which is to find out about the community’s rate of pregnancies, satisfaction with the HBC program, and knowledge of maternal health issues. Ask to interview women living in the household who are currently pregnant or were recently pregnant (between June 2012 and May 2014). Please interview each woman who lives in the household and has been pregnant over the observation period. Use one questionnaire per women (e.g., if two pregnant women live in the home, use two separate questionnaires).*

1. Date: _____________________________
2. Interviewer ID: _____________________
3. District: ___________________________
4. Ward name: ________________________
5. Street name: ________________________
6. House number: ______________________
7. Age of woman: ______________________
8. Name of woman: First: _________________ Middle:__________________ Last:_____________________
9. Other names of woman:___________________________________________________________________
10. RCH Card Number (4 digits):__________

***Request the woman’s RCH4 card. If she produces the card obtain the answers to the following questions directly from the card. If she does not produce the card, ask her to report the answers to the following questions.***

1. Is the RCH4 card available?

| *Select the best response with an X in left column* | | |
| --- | --- | --- |
|  | 1 | Yes |
|  | 2 | No |

1. Have you attended antenatal care during your most recent pregnancy between June 2012 and May 2014?

| *Select the best response with an X in left column* | | |
| --- | --- | --- |
|  | 1 | Yes |
|  | 2 | No |
|  | 3 | Unsure |

1. If yes, approximately how many months were you pregnant when you attended your first ANC visit?

| Number of months |  |
| --- | --- |

1. How many times did you attend ANC during this pregnancy?

| Number of ANC visits |  |
| --- | --- |

Please fill the dates of each of your ANC visits. Leave blank if not applicable.

|  | Date (day/month/year) |
| --- | --- |
| 1. ANC visit 1 |  |
| 1. ANC visit 2 |  |
| 1. ANC visit 3 |  |
| 1. ANC visit 4 |  |
| 1. ANC visit 5 |  |

1. What was the date of your last menstrual period?

| Date (day/month/year) |  |
| --- | --- |

1. Where do you intend to deliver?

| *Select the best response with an X in left column* | | |
| --- | --- | --- |
|  | 1 | At home **without** a birth attendant |
|  | 2 | At home **with** a birth attendant |
|  | 3 | At a healthcare facility |
|  | 4 | Upcountry |
|  | 5 | Other (specify): |

1. If you are no longer pregnant, what was the date of your delivery or miscarriage from your most recent pregnancy?

| Date (day/month/year) |  |
| --- | --- |

1. If you gave birth, where did you deliver?

| *Select the best response with an X in left column* | | |
| --- | --- | --- |
|  | 1 | At home **without** a birth attendant |
|  | 2 | At home **with** a birth attendant |
|  | 3 | At a healthcare facility |
|  | 4 | Other (specify): |

1. If you delivered at a health care facility, please give the name of the facility.

| Facility name |  |
| --- | --- |

1. What is the highest level of education you have attended?

| *Select the best response with an X in left column* | | |
| --- | --- | --- |
|  | 0 | Pre-primary |
|  | 1 | Primary |
|  | 2 | Post-primary |
|  | 3 | Secondary |
|  | 4 | Post-secondary |
|  | 5 | University |

1. How many adults (age 18 or older) live in your household?

| *Select the best response with an X in left column* | | |
| --- | --- | --- |
|  | 1 | 0 adults |
|  | 2 | 1 adult |
|  | 3 | 2 adults |
|  | 4 | 3 adults |
|  | 5 | 4 or more adults |

| *Please fill the table for each adult living in the household. Leave blank any that are not applicable.* | | | | | | | | |
| --- | --- | --- | --- | --- | --- | --- | --- | --- |
|  |  | Adult 1 | Adult 2 | Adult 3 | Adult 4 | Adult 5 | Adult 6 | Adult 7 |
|  | Gender (M or F) |  |  |  |  |  |  |  |
|  | Date of birth |  |  |  |  |  |  |  |

1. How many children (age 17 or younger) live in your household?___________________
2. Has your household ever been visited by a home-based carer (HBC)?

| *Select the best response with an X in left column* | | |
| --- | --- | --- |
|  | 1 | Yes |
|  | 2 | No (skip the next two questions) |
|  | 3 | Unsure |

1. How many times was the household visited by an HBC? ___________________ times
2. What is the approximate date of the last HBC visit? ______________________
3. Have you been pregnant at any time between June 2012 and May 2014?

| *Select the best response with an X in left column* | | |
| --- | --- | --- |
|  | 1 | No (do not continue survey) |
|  | 2 | Yes, a currently pregnant woman |
|  | 3 | Yes, a recently (since June 2012) pregnant woman |

1. How many women who live in your household have been pregnant since June 2012?

| *Select the best response with an X in left column* | | |
| --- | --- | --- |
|  | 1 | 0 pregnant women |
|  | 2 | 1 pregnant woman |
|  | 3 | 2 pregnant women |
|  | 4 | 3 pregnant women |
|  | 5 | 4 pregnant women or more |

1. How many women who live in the household gave birth (without miscarriage) since July 2012?

| *Select the best response with an X in left column* | | |
| --- | --- | --- |
|  | 1 | 0 women |
|  | 2 | 1 woman |
|  | 3 | 2 women |
|  | 4 | 3 women |
|  | 5 | 4 women or more |

1. How many women who live in the household had a miscarriage since July 2012?

| *Select the best response with an X in left column* | | |
| --- | --- | --- |
|  | 1 | 0 women (skip the next question) |
|  | 2 | 1 woman |
|  | 3 | 2 women |
|  | 4 | 3 women |
|  | 5 | 4 women or more |
|  | 6 | Don’t know |

***I would now like find out your level of knowledge of health issues for pregnant women.***

1. For your understanding; at how many months of pregnancy should a woman attend her first ANC visit?

| *Select the best response with an X in left column* | | |
| --- | --- | --- |
|  | 1 | Before 4 months |
|  | 2 | At 4-5 months |
|  | 3 | At 6-7 months |
|  | 4 | At 8-9 months |

1. How many times should a woman attend ANC during her pregnancy?

| *Select the best response with an X in left column* | | |
| --- | --- | --- |
|  | 1 | One time |
|  | 2 | Two times |
|  | 3 | Three times |
|  | 4 | Four times or more |

1. Is it possible for a baby to be born HIV-positive?

| *Select the best response with an X in left column* | | |
| --- | --- | --- |
|  | 1 | Yes |
|  | 2 | No |
|  | 3 | Don’t know |

1. If yes, what do you think are the reasons for which babies become infected with HIV? (tick all correct options)

| *Select the best response with an X in left column* | | |
| --- | --- | --- |
|  | 1 | Transmission from an HIV-positive mother who is not on ARV during pregnancy |
|  | 2 | Mosquito bites |
|  | 3 | Transmission from an HIV-positive mother who is not on ARV during breastfeeding |
|  | 4 | Not sleeping enough |
|  | 5 | Hot weather |
|  | 6 | Transmission from an HIV-positive mother who is not on ARV during delivery |

1. Where is it recommended for a pregnant woman to deliver? (tick the best option)

| *Select the best response with an X in left column* | | |
| --- | --- | --- |
|  | 1 | Alone at home |
|  | 2 | At home with a traditional birth attendant |
|  | 3 | At a health facility |
|  | 4 | Don’t know |

1. What feeding method is recommended for newborns for the first 6 months after delivery? (tick the best option)

| *Select the best response with an X in left column* | | |
| --- | --- | --- |
|  | 1 | Feeding of formula milk |
|  | 2 | Exclusive breastfeeding |
|  | 3 | Feeding of soft foods e.g. porridge, mashed potato |
|  | 4 | A mix of breastfeeding, formula milk, and/or soft foods |

1. Do you think pregnant women should get tested for HIV?

| *Select the best response with an X in left column* | | |
| --- | --- | --- |
|  | 1 | Yes |
|  | 2 | No |
|  | 3 | Undecided |

1. If yes, why?

| *Select the best response with an X in left column* | | |
| --- | --- | --- |
|  | 1 | If she has HIV, she can get medication during pregnancy to prevent transmission to the baby |
|  | 2 | If she has HIV, she may need medication to treat her HIV infection |
|  | 3 | If she has HIV, she can take measures to prevent her partner from getting infected |
|  | 4 | All of the above |

1. What was the date of your miscarriage?

| Date (day/month/year) |  |
| --- | --- |

1. Approximately how many months were you when the miscarriage occurred?

| Number of months |  |
| --- | --- |

1. Overall, how satisfied are you with the public health care system in Dar es Salaam?

*Select one option per woman.*

| *Select the best response with an X in left column* | | |
| --- | --- | --- |
|  | 1 | Very dissatisfied |
|  | 2 | Dissatisfied |
|  | 3 | Neutral |
|  | 4 | Satisfied |
|  | 5 | Very satisfied |

1. How satisfied are you with the HBC program?

| *Select the best response with an X in left column* | | |
| --- | --- | --- |
|  | 1 | Very dissatisfied |
|  | 2 | Dissatisfied |
|  | 3 | Neutral |
|  | 4 | Satisfied |
|  | 5 | Very satisfied |

***I would like to as a few questions concerning nutrition in your household.***

1. In the last 12 months, since July 2012, were you very hungry but didn't eat because there wasn't enough food in the household?

| *Select the best response with an X in left column* | | |
| --- | --- | --- |
|  | 1 | No |
|  | 2 | Yes |
|  | 3 | Don’t remember |

1. In the last 12 months (since July 2012) how many times did you **eat less than you felt you should** because there wasn't enough food in the household?

| *Select the best response with an X in left column* | | |
| --- | --- | --- |
|  | 1 | Never |
|  | 2 | Occasionally |
|  | 3 | Often |
|  | 4 | Nearly always |

1. In the last 12 months, since July 2012, did your **family ever cut the size of your meals** because there wasn't enough food in the household?

| *Select the best response with an X in left column* | | |
| --- | --- | --- |
|  | 1 | No |
|  | 2 | Yes |
|  | 3 | Not willing to answer |

1. In the last 12 months, since July 2012, did your **family ever skip meals** because there wasn't enough food in the household?

| *Select the best response with an X in left column* | | |
| --- | --- | --- |
|  | 1 | No |
|  | 2 | Yes |
|  | 3 | Not willing to answer |

1. If yes, how often did this happen?

| *Select the best response with an X in left column* | | |
| --- | --- | --- |
|  | 1 | Almost every month |
|  | 2 | Some months, but not every month |
|  | 3 | Not willing to answer |

1. *Please ask to see the salt used by the household in its original container and record whether the salt is:*

| *Select the best response with an X in left column* | | |
| --- | --- | --- |
|  | 1 | Not iodized |
|  | 2 | Iodized |

| *Please fill the table for* ***ALL*** *children 0-59 months in the household. Leave blank any that are not applicable.* | | | | | | | | |
| --- | --- | --- | --- | --- | --- | --- | --- | --- |
|  |  | Child 1 | Child 2 | Child 3 | Child 4 | Child 5 | Child 6 | Child 7 |
|  | Gender (M or F) |  |  |  |  |  |  |  |
|  | Date of birth |  |  |  |  |  |  |  |
|  | Weight |  |  |  |  |  |  |  |
|  | MUAC |  |  |  |  |  |  |  |
|  | Received Vitamin A in the past 12 months (Y or N) |  |  |  |  |  |  |  |

1. Think of the occasion you drank alcohol the most this past month (previous 30 days). How much did you drink?

| *Select the best response with an X in left column* | | |
| --- | --- | --- |
|  | 1 | Zero (0) drinks |
|  | 2 | One (1) drink |
|  | 3 | Two (2) drinks |
|  | 4 | Three (3) drinks |
|  | 5 | Four (4) drinks |
|  | 6 | More than four drinks |

1. Think of a time you **wanted to drink** alcohol this past month (previous 30 days). How strong was your desire to drink?

| *Select the best response with an X in left column* | | |
| --- | --- | --- |
|  | 1 | I did not have any desire to drink at all |
|  | 2 | My desire to drink was very weak |
|  | 3 | My desire to drink was somewhat weak |
|  | 4 | My desire to drink was somewhat strong |
|  | 5 | My desire to drink was very strong |

1. How often did you drink alcohol during the past month (previous 30 days)?

| *Select the best response with an X in left column* | | |
| --- | --- | --- |
|  | 1 | I do not drink at all |
|  | 2 | Once a month |
|  | 4 | Several times per month |
|  | 5 | Once a week |
|  | 6 | Several times per week |
|  | 7 | Every day |
|  | 8 | Several times per day |

***I would like to ask a few questions on cervical cancer.***

1. Have you ever been ***screened*** for cervical cancer?

| *Select the best response with an X in left column* | | |
| --- | --- | --- |
|  | 1 | No (skip next question) |
|  | 2 | Yes |

1. If yes, how were you ***screened*** for it, if at all?

| *Select the best response with an X in left column* | | |
| --- | --- | --- |
|  | 1 | Pap test |
|  | 2 | HPV test |
|  | 3 | Other (please specify): |

1. What was the outcome of the test?

| *Select the best response with an X in left column* | | |
| --- | --- | --- |
|  | 1 | Results were normal |
|  | 2 | Results were abnormal |
|  | 3 | I do not know |

1. If cervical cancer screening were to be made available to you, would you like to be screened?

| *Select the best response with an X in left column* | | |
| --- | --- | --- |
|  | 1 | Yes |
|  | 2 | No |
|  | 3 | Undecided |

***I would like to ask a few questions about gestational diabetes.***

1. Have you ever been diagnosed with gestational diabetes?

| *Select the best response with an X in left column* | | |
| --- | --- | --- |
|  | 1 | Yes |
|  | 2 | No (skip the next 2 questions) |

1. If you have ever been diagnosed with gestational diabetes, how were you treated?

| *Select the best response with an X in left column* | | |
| --- | --- | --- |
|  | 1 | Insulin |
|  | 2 | Glyburide |
|  | 3 | Diet controlled only |
|  | 4 | Other (specify): |
|  | 5 | No treatment |

1. Do you have a meal plan for diabetes?

| *Select the best response with an X in left column* | | |
| --- | --- | --- |
|  | 1 | Calorie counting |
|  | 2 | Food pyramid / healthy choices |
|  | 3 | Low carbohydrate |
|  | 4 | No added sugar |
|  | 5 | Other (specify) |
|  | 6 | No meal plan for diabetes |

***I would like to ask a few questions about hypertension and depression.***

1. Have you ever been diagnosed with high blood pressure?

| *Select the best response with an X in left column* | | |
| --- | --- | --- |
|  | 1 | Yes |
|  | 2 | No (skip the next question) |

1. Please provide the date range when high blood pressure was last diagnosed?

| *Select the best response with an X in left column* | | |
| --- | --- | --- |
|  | 1 | Within the past month |
|  | 2 | Within the past 6 months |
|  | 3 | More than 6 months ago |

1. Have you taken prescribed medication for high blood pressure within the past year (12 months)?

| *Select the best response with an X in left column* | | |
| --- | --- | --- |
|  | 1 | Yes |
|  | 2 | No |

|  | ***Please select the best response for each item.*** | Not at all | Just a little | Somewhat | Moderately | Quite a lot | Very much |
| --- | --- | --- | --- | --- | --- | --- | --- |
|  | I do things slowly |  |  |  |  |  |  |
|  | My future seems hopeless |  |  |  |  |  |  |
|  | It is hard for me to concentrate on reading |  |  |  |  |  |  |
|  | The pleasure and joy has gone out of my life |  |  |  |  |  |  |
|  | I have difficulty making decisions |  |  |  |  |  |  |
|  | I have lost interest in aspects of life that used to be important to me |  |  |  |  |  |  |
|  | I feel sad, blue, and unhappy |  |  |  |  |  |  |
|  | I am agitated and keep moving around |  |  |  |  |  |  |
|  | I feel fatigued |  |  |  |  |  |  |

***I would like to ask a few questions about contraception.***

1. Did you use anything or tried in any way to delay or avoid getting pregnant before this pregnancy?

| *Select the best response with an X in left column* | | |
| --- | --- | --- |
|  | 1 | Yes |
|  | 2 | No |

1. If yes, what methods did you use before this pregnancy?

|  | *Please indicate YES or NO for each item by placing an X in a column on the right.* | Yes | No |
| --- | --- | --- | --- |
|  | Injectables |  |  |
|  | Pill |  |  |
|  | Male Condom |  |  |
|  | Rhythm method |  |  |
|  | Female sterilization |  |  |
|  | Withdrawal |  |  |
|  | Implants |  |  |
|  | Lactational amenorrhea method |  |  |
|  | IUD |  |  |
|  | Male sterilization |  |  |
|  | Diaphragm |  |  |
|  | Foam/Jelly |  |  |
|  | CycleBeads |  |  |
|  | Other modern method |  |  |
|  | Other traditional method |  |  |

1. Do you plan to use any method to delay or avoid getting pregnant after the birth of this baby?

| *Select the best response with an X in left column* | | |
| --- | --- | --- |
|  | 1 | Yes |
|  | 2 | No (skip to end) |

1. How long after the birth of this baby do you plan to start using any method to delay or avoid getting pregnant?__________________________________________
2. What methods do you plan to use after the birth of this baby to delay or avoid getting pregnant?

|  | *Please indicate YES or NO for each item by placing an X in a column on the right.* | Yes | No |
| --- | --- | --- | --- |
|  | Injectables |  |  |
|  | Pill |  |  |
|  | Male Condom |  |  |
|  | Rhythm method |  |  |
|  | Female sterilization |  |  |
|  | Withdrawal |  |  |
|  | Implants |  |  |
|  | Lactational amenorrhea method |  |  |
|  | IUD |  |  |
|  | Male sterilization |  |  |
|  | Diaphragm |  |  |
|  | Foam/Jelly |  |  |
|  | CycleBeads |  |  |
|  | Other modern method |  |  |
|  | Other traditional method |  |  |

1. If you are planning to delay or avoid getting pregnant after the birth of this baby, do you know where to get this method (if applicable)?

| *Select the best response with an X in left column* | | |
| --- | --- | --- |
|  | 1 | Yes |
|  | 2 | No (skip to end) |

Where will you go to get this method? Leave blank any that are not applicable.

|  | *Select all that apply by placing an X in a column on the right.* |  |
| --- | --- | --- |
|  | Government hospital |  |
|  | Government health center |  |
|  | Family planning clinic |  |
|  | Mobile clinic |  |
|  | Fieldworker |  |
|  | Other public sector |  |
|  | Private hospital/clinic |  |
|  | Pharmacy |  |
|  | Private Doctor |  |
|  | Shop |  |
|  | Church |  |
|  | Friend/relative |  |
|  | Other |  |

**Please thank the interviewee for her time.**
