# Supplementary material for: Community health workers to improve uptake of maternal healthcare services: A cluster-randomized pragmatic trial in Dar es Salaam, Tanzania
Source: PLoS Med. 2019 Mar 29;16(3):e1002768. doi: 10.1371/journal.pmed.1002768 (PMC6440613; doi:10.1371/journal.pmed.1002768)
Supplement: S2 Text — (DOCX) [file pmed.1002768.s013.docx]

## **Text S2. Patient satisfaction questionnaire**

| *Management and Development for Health (MDH) is conducting a study in Dar es Salaam to improve antenatal and PMTCT services. As part of this study, we would like to find out patients’ experience of their care at antenatal and PMTCT clinics. The completion of the survey is voluntary (your choice) and all responses will be kept private and not shared with the health care providers in a way that you could be identified. If you do not want to do this survey, it will not affect your care at this or any other health facility now or in the future. It should take approximately 30 minutes to complete.* |
| --- |

1. Date of interview: ___________
2. Time at the start and end of the interview: Start: __________ End: __________
3. Facility name: _____________________
4. Name of the interviewer: _____________________________

5a) What services did you come for today?

| First antenatal care (ANC) visit | 1 |
| --- | --- |
| Follow-up ANC visit (scheduled) | 2 |
| ARV prophylaxis/ART (refill) | 3 |
| Infant HIV testing | 4 |
| Infant follow-up visit | 5 |
| Sick visit | 6 |
| Other | 7 |
| If other, please specify | _______________________ |

5b) If the patient did not come for “ARV prophylaxis/ART (refill): Have you ever attended a visit for ARV prophylaxis/ART (refill) during this pregnancy?

| Yes | 1 |
| --- | --- |
| No | 2 |

1. How would you rate the overall healthcare that you received today? *(Circle one appropriate response)*

| Very good | 1 |
| --- | --- |
| Good | 2 |
| Moderately good | 3 |
| Fair | 4 |
| Poor | 5 |

1. Overall, how would you rate your experience of how well health care providers communicated with you during this visit?

*[Good communication is when the provider listens to you carefully, explains things so you can understand, and you have time to ask questions.]* (*Circle one appropriate response)*

| Very good | 1 |
| --- | --- |
| Good | 2 |
| Moderately good | 3 |
| Fair | 4 |
| Poor | 5 |

1. Who was the health care provider who provided most of care to you today?

| Doctor | 1 |
| --- | --- |
| Nurse | 2 |
| Counselor | 3 |
| Pharmacist | 4 |
| Others  If others, please specify--- | 5 |

1. How did you find the health care provider listened to you during your visit? (*Circle one appropriate response)*

| Listened very well | 1 |
| --- | --- |
| Listened well | 2 |
| Listened moderately well | 3 |
| Only listened a little | 4 |
| Didn’t listen at all | 5 |

1. Did the health care provider explain things in a way you could understand during this visit?

| Most of the time | 1 |
| --- | --- |
| Sometimes | 2 |
| No | 3 |

1. Did the health care provider give you time to ask question this visit?

| Most of the time | 1 |
| --- | --- |
| Sometimes | 2 |
| No | 3 |

1. Overall how well do you feel the health care providers you saw understood your health concerns during this clinic visit? (*Circle one appropriate response)*

| Very well | 1 |
| --- | --- |
| Well | 2 |
| Moderately well | 3 |
| Fair | 4 |
| Poorly/Not well at all | 5 |

1. Overall, how would you rate your experience of the way the clinic kept information about you confidential?

[*Confidentiality of information means having your medical history kept confidential, and having talks with health providers done so that other people cannot listen in.]* (*Circle one appropriate response)*

| Very good | 1 |
| --- | --- |
| Good | 2 |
| Moderately good | 3 |
| Fair | 4 |
| Poor | 5 |

1. Overall, how would you rate the promptness of attention at this clinic today?

*[Prompt attention means that you have short waiting times for appointments and get tests done quickly.]* (*Circle one appropriate response)*

| Very good | 1 |
| --- | --- |
| Good | 2 |
| Moderately good | 3 |
| Fair | 4 |
| Poor | 5 |

1. How were the technical skills of health care providers? (*Circle one appropriate response)*

| Very good | 1 |
| --- | --- |
| Good | 2 |
| Moderately good | 3 |
| Fair | 4 |
| Poor | 5 |

16a) If you were prescribed or had to pick-up any medication today, were you able to get them?

| Yes | 1 |
| --- | --- |
| No | 2 |
| I was not prescribed any medication today | 3 |

16b) If no, why not? (Tick all that apply)

| Drugs were not available at the pharmacy | 1 |
| --- | --- |
| The queue was too long so I left | 2 |
| The pharmacy was closed or pharmacist was not available | 3 |
| Other, describe | 4 |
| Don’t know | 5 |

17a. ) Were the services offered in this clinic meet your expectations?

| Yes | 1 |
| --- | --- |
| No | 2 |
| Not sure | 3 |

17.b) Would you recommend your friend or relative who is pregnant to attend this facility?

| Yes | 1 |
| --- | --- |
| No | 2 |
| Not sure | 3 |

1. Are the opening hours of this clinic convenient for you?

| Yes | 1 |
| --- | --- |
| No | 2 |

*We would now like to ask you a few questions to find out how difficult it is for you to take time off to come to the clinic.*

1. How long did it take to arrive at the clinic today? *(Circle one appropriate response)*

| Less than 15 minutes | 1 |
| --- | --- |
| 15-30 minutes | 2 |
| 31-60 minutes | 3 |
| More than one hour | 4 |

1. How difficult is it for you to get to the clinic?  *(Circle one appropriate response)*

| Very difficult | 1 |
| --- | --- |
| Difficult | 2 |
| Moderately difficult | 3 |
| Easy | 4 |
| Very easy | 5 |

21a) Have you ever missed an ANC or PMTCT appointment during this pregnancy?

| Yes | 1 |
| --- | --- |
| No | 2 skip b |
| Not sure | 3 skip b |

21b) If yes, what was the reason for missing the ANC or PMTCT appointment? *(Check all that apply)*

| I had no money for transport | 1 |
| --- | --- |
| I had no time to come for my appointment | 2 |
| I was not feeling well | 3 |
| I could not get permission to leave work | 4 |
| The quality of service is poor | 5 |
| Other | 6 |
| If other, please specify | ________________________ |

1. Are you covered by a scheme, such as an insurance, that helps you pay for healthcare?

| Yes | 1 |
| --- | --- |
| No | 2 |

22a) Have you ever spent money on healthcare in this clinic in the past month?

| Yes | 1 |
| --- | --- |
| No | 2 skip b |

22b) If yes, what type of expenses did you pay for and the amount? *(Fill in the amount spent for each)*

| Consultation fee | __________________TSh |
| --- | --- |
| Investigations | __________________TSh |
| Medicine | __________________TSh |
| Others | __________________TSh |

1. In coming to ANC or PMTCT today, how much did you pay for: (tick all that apply)

| Transport (one way) | __________________TSh |
| --- | --- |
| Including child-care | __________________TSh |
| Food during visit | __________________TSh |
| Phone call/SMS | __________________TSh |
| Other | __________________TSh |

1. Did you find it easy or difficult to incur these expenses? (*Circle one appropriate response*)

| Easy | 1 |
| --- | --- |
| Difficult | 2 |
| Neither easy nor difficult | 3 |
| Don’t know | 4 |

25a) In the last month did you have to borrow money to pay for health services in this facility?

| Yes | 1 |
| --- | --- |
| No | 2 skip b |

25b) If yes, how much money did you borrow?____________________ TSh

1. In the last month did you have to sell personal or household items in order to pay for health services in this facility?

| Yes | 1 |
| --- | --- |
| No | 2 |

1. How much time did you spend at the clinic last time you came for ANC or PMTCT?_____ hours _______minutes ( from the time you arrived to the facility to the time you left?
2. How much time does it take for you to get from your home to the ANC or PMTCT clinic?_____ _____ hours _________ minutes

29a) What would you have been doing if you weren’t at the clinic today? (*Circle all that apply*)

| Working for pay | 1 |
| --- | --- |
| Doing unpaid community work or volunteer work | 2 |
| Doing household chores such as cleaning, cooking, shopping for food, maintenance and repairs, working in the garden, gathering wood, gathering water, housework, etc | 3 |
| Taking care of children | 4 |
| Leisure activities (sport, watching TV, listening to music, reading, visiting friends and family, going to movies, etc.) | 5 |
| Attending school or other educational institution | 6 |
| Nothing | 7 |
| I don’t know | 8 |
| Other, specify | 9 |

29b) If you are working for pay, did you lose income from the time you took from your job to come here today?

| Yes | 1 |
| --- | --- |
| No | 2 *skip option c* |

29c) If yes, how much money did you lose?_________________ TSh

1. Does attending ANC interfere with your ability to fulfill your role in your family?

| Yes | 1 |
| --- | --- |
| No | 2 |
| Uncertain | 3 |
|  |  |

**If the patient has not attended a PMTCT or infant HIV-testing visit during this pregnancy, skip to question 41.**

*We would now like to ask you a few questions about your experience with your HIV infection.*

| To what extent do you disagree or agree with the following statements? | **Strongly disagree** | **Disagree** | **Neutral** | **Agree** | **Strongly**  **Agree** |
| --- | --- | --- | --- | --- | --- |
| 1. I am overwhelmed by the PMTCT visit schedule | ☐ | ☐ | ☐ | ☐ | ☐ |
| 1. I don’t have any problems in adhering to my PMTCT medications | ☐ | ☐ | ☐ | ☐ | ☐ |
| 1. I am able to cope with my HIV infection | ☐ | ☐ | ☐ | ☐ | ☐ |

1. When did you find out if you were HIV positive?

_______________ ____________________

MM YYYY

1. When did you FIRST begin receiving antiretroviral (ARV) treatment?

_______________ ____________________

MM YYYY

36a) Have you disclosed your HIV serostatus?

| Yes | 1 |
| --- | --- |
| No | 2 |

36b) If yes, who did you disclose your HIV status to when you learned that you are HIV positive? *(circle all that apply)*

| Spouse or steady sexual partner | 1 |
| --- | --- |
| Child | 2 |
| Mother | 3 |
| Other female relative | 4 |
| Father | 5 |
| Other male relative | 6 |
| Sister | 7 |
| Friend | 8 |
| Brother | 9 |
| Other | 10 |
| If other, please specify | ________________________ |

1. Were you ever worried that attending PMTCT or taking PMTCT medications may disclose your HIV status to people who you do not want to know about your HIV infection?

| Yes | 1 |
| --- | --- |
| No | 2 |
| Uncertain | 3 |

1. Has attending PMTCT or taking PMTCT medications actually caused a disclosure of your HIV status to people who you do not want to know about your HIV infections?

| Yes | 1 |
| --- | --- |
| No | 2 |
| Uncertain | 3 |

1. Are you worried that you may experience any of the following social problems from attending PMTCT visits or taking PMTCT medications? (*Circle all that apply*)

| Conflict with spouse or partner | 1 |
| --- | --- |
| Separation or divorce from spouse or partner | 2 |
| Abandonment by spouse or partner | 3 |
| Beating or other forms of physical violence by spouse or partner | 4 |
| Isolation and/or lack of support from family or friends | 5 |
| Being a burden or source of worry for others | 6 |
| Teasing or insulting | 7 |
| Loss of respect or standing with the family and/or community | 8 |
| Loss of customers | 9 |
| Loss of a job | 10 |
| Taking away of property | 11 |
| Taking away of a child | 12 |

1. Have you actually experienced any of the social problems listed in question 52?

| Yes | 1 |
| --- | --- |
| No | 2 |
| If yes, which problem did you experience | __________________________ |

*We would now like to ask you some questions about your knowledge on maternal health and infant feeding issues.*

1. What feeding method is recommended for newborns for the first 6 months after delivery? (*Circle only the most correct option*)

| Feeding of formula milk | 1 |
| --- | --- |
| Exclusive breastfeeding | 2 |
| Feeding of soft foods e.g. porridge, mashed potato | 3 |
| A mix of breastfeeding, formula milk, and/or soft foods | 4 |

1. Where is it recommended for a pregnant woman to deliver? (*Circle only the most correct option*)

| Alone at home | 1 |
| --- | --- |
| At home with a traditional birth attendant | 2 |
| At a health facility | 3 |
| Undecided | 4 |

1. a) Is it possible for a baby to be HIV-positive?

| Yes | 1 |
| --- | --- |
| No | 2 |
| Undecided | 3 |

1. b) If yes, what do you think are the reasons for which babies become infected with HIV? (*circle all correct options*)

| Transmission from an HIV-positive mother during pregnancy | 1 |
| --- | --- |
| Mosquito bites | 2 |
| Transmission from an HIV-positive mother during breastfeeding | 3 |
| Skipping ARV prophylaxis | 4 |
| Breastfeeding and giving other feeds | 5 |
| Transmission from an HIV-positive mother during delivery | 6 |

**Ask questions 45-48 only if the patient came for a PMTCT visit or infant HIV-testing.**

1. ARVs cure HIV/AIDS.

| True/Correct | 1 |
| --- | --- |
| False/Incorrect | 2 |
| Don’t know | 3 |

1. If you got your medications today, were you told how to take them?

| Yes | 1 |
| --- | --- |
| No | 2 |

1. How many times a day should you take your medications?

| Once | 1 |
| --- | --- |
| Twice | 2 |
| Three or more times | 3 |

1. Were possible side effects or complications of the medications explained?

| Yes | 1 |
| --- | --- |
| No | 2 |

*We would now like to ask you some questions about yourself. No attempt will be made to identify individual people from these answers.*

1. What is your age?  years
2. How many times have you given birth? _______ _________ time
3. What is your level of formal education? (*Circle one appropriate response*)

| Not been to school | 1 |
| --- | --- |
| Primary Education | 2 |
| Secondary Education (form I-IV) | 3 |
| Secondary Education (form V-VI) | 4 |
| Certificate or diploma training | 5 |
| University | 6 |

1. What is your marital status? (*Circle one appropriate response*)

| Married | 1 |
| --- | --- |
| Divorced or separated | 2 |
| Living with partner | 3 |
| Never married/single | 4 |
| Widowed | 5 |
| Other | 6 |
| If other, please specify | _________ |

1. What is your current occupation?

| Unemployed | 1 |
| --- | --- |
| Employed | 2 |
| Self-employed | 3 |
| Housewife | 4 |
| Other | 5 |
| If other, please specify | _________ |

1. In the house where you are living, do you have the following items? (*Circle all correct options*)

| Radio | 1 |
| --- | --- |
| Bicycle | 2 |
| Electricity | 3 |
| Running water | 4 |
| TV | 5 |
| Car | 6 |
| Mobile phone | 7 |

1. How would you describe where you live?

| I or my family own the house | 1 |
| --- | --- |
| Stay in a rented house | 2 |
| No regular place to stay | 3 |
| Other | 4 |
| If other, please specify | _________ |

1. Where you are living, is the house made of concrete/brick?

| Yes | 1 |
| --- | --- |
| No | 2 |

1. How many adults are there in your household?______________ adults
2. How many children are there in your household? __________ Children

59a) In general how much does your household usually spend in a month? --------TSh

59b) *If the respondent does not give you a precise estimate, ask him/her:*

In which of the following ranges, would you say your household EXPENDITURE generally falls? (*Circle one appropriate response)*

| Less than 50,000 TSh | 1 |
| --- | --- |
| 50,000 – 100,000 TSh | 2 |
| 101,000 - 300,000 TSh | 3 |
| 301,000 - 500,000 TSh | 4 |
| More than 500,000 TSh | 5 |

1. Assume that you do not yet need ARV drugs for your own health, because you have a high CD4 count and do not yet have any symptoms of HIV. If you were given different alternatives to prevent your baby from becoming infected with HIV that were all equally effective and safe for the baby, and you had to choose one option, which of the following options would you prefer? *(Circle the response)*

| **Option A:** Taking one ARV (AZT) before birth starting as early as 14 weeks of gestation, taking different ARV drugs at onset of labor and daily for 7 days after giving birth. In addition, your baby would receive one ARV drug (NVP) every day after it has been born and during the breastfeeding period, or for 4-6 weeks if you are not breastfeeding. | 1 | |
| --- | --- | --- |
| **Option B:** Taking three ARV drugs during pregnancy starting as early as 14 weeks of gestation, and through childbirth and breastfeeding, and then stop taking the medication. In addition, your baby would receive one ARV drug (NVP or AZT) after it has been born for 4-6 weeks, regardless of whether you are breastfeeding or not. | 2 |  |
| **Option B+:** Taking the ARV drugs yourself during pregnancy and breastfeeding and then continue taking the medication for life. In addition, your baby would receive one ARV drug (NVP or AZT) after it has been born for 4-6 weeks, regardless of whether you are breastfeeding or not. | 3 |  |
| **I don’t know** | 4 |  |

If you want, please explain why you prefer the option

|  |
| --- |

1. B. What method are you recently using to protect your child from acquiring HIV infection?

| Option A | 1 |
| --- | --- |
| Option B | 2 |
| Option B+ | 3 |
| I don’t know | 4 |

60.C If you are using option option B+, when did you start to use this option?

| One week ago | 1 |
| --- | --- |
| Two weeks ago | 2 |
| One month ago | 3 |
| More than one month | 4 |

60.D Are there any difficulties which you faced to change from the method you were using before? moving to option B+

| Yes | 1 |
| --- | --- |
| No | 2 |

**Thank you very much for your effort and time!**

**PLEASE LET US KNOW IF YOU HAVE ANY COMMENTS ON THIS QUESTIONNAIRE**

|  |
| --- |
